# ngLOC: an *n*-gram-based Bayesian method for estimating the subcellular proteomes of eukaryotes

**DOI:** 10.1186/gb-2007-8-5-r68

**Published:** 2007-05-01

**Authors:** Brian R King, Chittibabu Guda

**Affiliations:** 1 Department of Computer Science, State University of New York at Albany, Washington Ave, Albany, New York 12222, USA; 2 Gen*NY*sis Center for Excellence in Cancer Genomics, State University of New York at Albany, Discovery Drive, Rensselaer, New York 12144-3456, USA; 3 Department of Epidemiology and Biostatistics, State University of New York at Albany, Discovery Drive, Rensselaer, New York 12144-3456, USA

## Abstract

We present a method called ngLOC, an *n*-gram-based Bayesian classifier that predicts the localization of a protein sequence over ten distinct subcellular organelles. A tenfold cross-validation result shows an accuracy of 89% for sequences localized to a single organelle, and 82% for those localized to multiple organelles. An enhanced version of ngLOC was developed to estimate the subcellular proteomes of eight eukaryotic organisms: yeast, nematode, fruitfly, mosquito, zebrafish, chicken, mouse, and human.

## Abstract 2

ngLOC is an *n*-gram-based Bayesian classification method that can predict the localization of a protein sequence over ten distinct subcellular organelles.

## Background

Subcellular or organellar proteomics has gained tremendous attention of late, owing to the role played by organelles in carrying out defined cellular processes. Several efforts have been made to catalog the complete subcellular proteomes of various model organisms (for review [1,2]), with the aim being to improve our understanding of defined cellular processes at the organellar and cellular levels. Although such efforts have generated valuable information, cataloging all subcellular proteomes is far from complete. Experimental methods can be expensive, often generating conflicting or inconclusive results because of inherent limitations in the methods [3,4]. To complicate matters, computational methods rely on these experimental data, and therefore they must be resilient to noisy or inconsistent data found in these large datasets. These dilemmas have made the task of obtaining the complete set of proteins for each subcellular organelle a highly challenging one.

In this study we address the task of estimating the subcellular proteome through development of a computational method that can be used to annotate the subcellular localization of proteins on a proteomic scale. A fundamental goal of computational methods in bioinformatics research is to annotate newly discovered protein sequences with their functional information more efficiently and accurately. Protein subcellular localization prediction has become a crucial part of establishing this important goal. In this task, predictive models are inferred from experimentally annotated datasets containing subcellular localization information, with the objective being to use these models to predict the subcellular localization of a protein sequence of unknown localization.

The methods developed for predicting subcellular localization have varied significantly, ranging from the seminal work by Nakai and Kanehisa [5] on PSORT, which is a rule-based system derived by considering motifs and amino acid compositions; to the pure statistics based methods of Chou and Elrod [6], which employed covariant discriminant analysis; to the numerous methods available today, which are based on a variety of machine learning and data mining algorithms, including artifical neural networks and support vector machines (SVMs) [7,8]. All methods must choose a set of features to represent a protein in the classification system. Although the majority of methods use various facets of information derived from the sequence, others use phylogenic information [9], structure information [10], and known functional domains [11]. Some methods scan documents and annotations related to the proteins in their dataset in search of discriminative keywords that can be used as predictive indicators [12,13]. Regardless of the representation, the sequence of a protein contains virtually all of the information needed to determine the structure of the protein, which in turn determines its function. Therefore, it is theoretically possible to derive much of the information needed to resolve most protein classification problems directly from the protein sequence. Furthermore, it has been proposed that a significant relationship exists between sequence similarity and subcellular localization [14], and the majority of protein classification methods have capitalized on this assumption.

In addition to different classification algorithms and protein representation models, subcellular localization prediction methods also differ in exactly what they classify. Some consider only one or a few organelles in the cell [15,16]. Others consider all of the major organelles [5,6,8,11]. Methods often limit the species being considered, such as the PSORTb classifier for gram-negative bacteria [17]. Others limit the type of proteins being considered, such as those related to apoptosis [18]. We refer the interested reader to a review by Dönnes and Höglund [19], which provides an overview of the various methods used in this vast field.

High-throughput proteomic studies continue to generate an ever-increasing quantity of protein data that must be analyzed. Hence, computational methods that can accurately and efficiently elucidate these proteins with respect to their functional annotation, including subcellular localization, at the level of the proteome are urgently needed [20]. Although a variety of computational methods are available for this task, very few of them have been applied on a proteome-wide scale. The PSLT method [21], a Bayesian method that uses a combination of InterPro motifs, signaling peptides, and human transmembrane domains, was used to estimate the subcellular proteome on portions of the proteome of human, mouse, and yeast. The method of Huang and Li [22], a fuzzy *k*-nearest neighbors algorithm that uses dipeptide compositions obtained from the protein sequence, was used to estimate the subcellular proteome for six species over six major organelles.

Despite the availability of an array of methods, most of these are not suitable for proteome-wide prediction of subcellular localization for the following reasons. First, most methods predict only a limited number of locations. Second, the scoring criteria used by most methods are limited to subsets of proteomes, such as those containing signal/target peptide sequences or those with prior structural or functional information. Third, the majority of methods predict only one subcellular location for a given protein, even though a significant number of eukaryotic proteins are known to localize in multiple subcellular organelles. Fourth, many methods exhibit a lack of a balance between sensitivity and specificity. Fifth, the datasets used to train these programs are not sufficiently robust to represent the entire proteomes, and in some cases they are outdated or altered. Finally, many methods require the use of additional information beyond the primary sequence of the protein, which is often not available on a proteome-wide scale.

In this report we present ngLOC, a Bayesian classification method for predicting protein subcellular localization. Our method uses *n*-gram peptides derived solely from the primary structure of a protein to explore the search space of proteins. It is suitable for proteome-wide predictions, and is also capable of inferring multi-localized proteins, namely those localized to more than one subcellular location. Using the ngLOC method, we have estimated the sizes of ten subcellular proteomes from eight eukaryotic species.

## Results

We use a naïve Bayesian approach to model the density distributions of fixed-length peptide sequences (*n*-grams) over ten different subcellular locations. These distributions are determined from protein sequence data that contain experimentally determined annotations of subcellular localizations. To evaluate the performance of the method, we apply a standard validation technique called tenfold cross-validation, in which sequences from each class are divided into ten parts; the model is built using nine parts, and predictions are generated and evaluated on the data contained in the remaining part. This process is repeated for all ten possible combinations. We report standard performance measures over each subcellular location, including sensitivity (recall), precision, specificity, false positive rate, Matthews correlation coefficient (MCC), and receiver operating characteristic (ROC) curves. MCC provides a measure of performance for a single class being predicted; it equals 1 for perfect predictions on that class, 0 for random assignments, and less than 0 if predictions are worse than random [23]. For a measure of the overall classifier performance, we report overall accuracy as the fraction of the data tested that were classified correctly. (All of our formulae used to measure performance are briefly explained in the Materials and methods section [see below], with details provided in Additional data file 1.) To demonstrate the usefulness of our probabilistic confidence measures, we show how these measures can be used to consider situations in which a sequence may have multiple localizations, as well as to consider alternative localizations when confidence is low.

### Evaluation of different size *n*-grams

**Figure 1:**
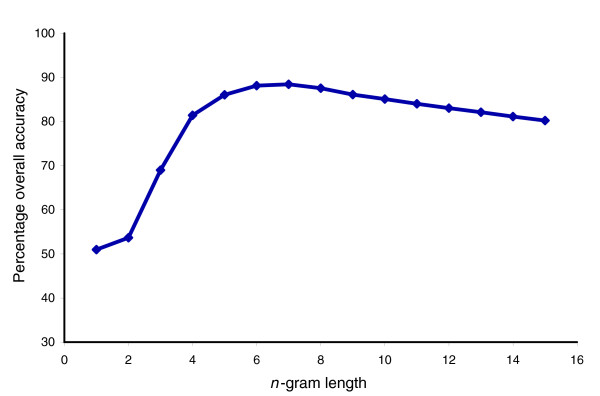
Overall accuracy versus *n*-gram length. This graph shows how different values of *n *affect the overall accuracy of ngLOC on our dataset. We define percentage overall accuracy as the percentage of data that were predicted with the correct localization, based on a tenfold cross-validation.

In the context of proteins, an *n*-gram is defined as a subsequence of the primary structure of a protein of a fixed-length size of *n*. First, we determined the optimal value of *n *to use by evaluating the predictive performance of ngLOC over different size *n*-gram models up to 15-grams. For this test only, we used only single-localized sequences, and set the minimum allowable length sequence to be 15 to enable testing of models up to 15-grams. Our results show that the 7-gram model had the highest performance, with an overall accuracy of 88.43%. However, both the 6-gram and 8-gram models are close to this level of performance, with accuracies of 88.12% and 87.53%, respectively (Figure 1). The results reported in the rest of this report use the 7-gram model, unless otherwise stated.

### Prediction performance using a 7-gram model

All of our tests are based on the standard ngLOC dataset (detailed in the Materials and methods section [see below]), which was selected with a minimum sequence length of 10 residues allowed. We ran a test using only single localized sequences, as well as the entire dataset including multi-localized sequences. For a 7-gram model, the overall accuracy of both models on single-localized sequences only was 88.8% and 89%, respectively. The results for the model built using the entire dataset is shown in Table 1, and will be the model of choice because it will enable prediction of multi-localized sequences as well.

**Table 1:** Results for 7-gram model using entire dataset

Location	Code	Precision	Sensitivity	FPR	Specificity	MCC
Cytoplasm	CYT	0.828	0.775	0.020	0.980	0.777
Cytoskeleton	CSK	0.882	0.452	0.001	0.999	0.629
Endoplasmic Reticulum	END	0.961	0.789	0.001	0.999	0.867
Extracellular	EXC	0.949	0.939	0.021	0.979	0.921
Golgi Apparatus	GOL	0.891	0.550	0.001	0.999	0.697
Lysosome	LYS	0.953	0.855	0.000	1.000	0.902
Mitochrondria	MIT	0.964	0.799	0.003	0.997	0.867
Nuclear	NUC	0.807	0.906	0.048	0.952	0.821
Plasma Membrane	PLA	0.883	0.958	0.043	0.957	0.892
Perixosome	POX	0.938	0.748	0.000	1.000	0.836

Single-localized % overall accuracy						89.03
Multi-localized % overall accuracy (at least 1 correct)						81.88
Multi-localized % overall accuracy (both correct)						59.70

The performance results of ngLOC on a tenfold cross-validation are displayed. The overall accuracy is also reported for multi-localized sequences, comparing at least one localization predicted correctly against both localizations predicted correctly. FPR, false positive rate; MCC, Matthews correlation coefficient.

Referring to Table 1, precision is high across all classes (0.81 to 0.96), whereas sensitivity ranged between 0.75 to 0.96, with the exception of golgi (GOL; 0.55) and cytoskeleton (CSK; 0.45), which is probably due to low representation in the dataset. Although CSK and GOL had the lowest sensitivity, their precision was very good, which is typical when a class is under-predicted. Specificity is very high across all classes (0.95 to 1.0), although the classes with the largest representation in the dataset, namely extracellular (EXC), plasma membrane (PLA), nuclear (NUC), and cytoplasm (CYT), had the lowest specificity, which is typical for highly represented classes that are often prone to over-prediction. Regardless, the MCC values for these four classes were still between 0.78 and 0.92. On the other end are the classes with the smallest representations in the dataset, including lysosome (LYS), peroxisome (POX), CSK, and GOL, whose MCC values range between 0.63 and 0.90. Surprisingly, LYS and POX, the two classes with the smallest representation in the dataset, had good MCC values (0.902 and 0.836, respectively). We determined the percentage of *n*-grams that were unique (occurred in only one organelle) in each of these four organelles (LYS, POX, CSK, and GOL) and discovered that LYS and POX had the highest percentage of unique *n*-grams with respect to the total number of *n*-grams in the organelle (data not shown). This suggests that the proteins in these locations are highly specific and distinctive compared with those proteins localized elsewhere, and could explain the superior performance of these locations despite their having the smallest representation in the training dataset. We also observed that *n*-grams in CSK and GOL had the lowest percentage of unique *n*-grams compared with any other class in the data, suggesting that *n*-grams in these organelles are more likely to be in common with *n*-grams in other organelles, and therefore the proteins in these organelles will be difficult to predict. The remaining classes performed well, with MCC values of 0.87.

**Figure 2:**
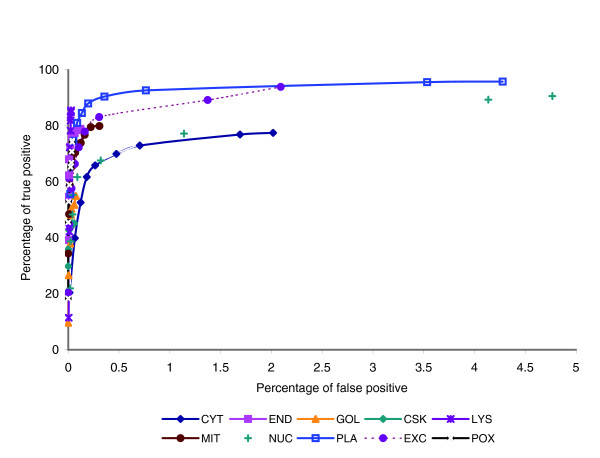
ROC curve for 7-gram model. A plot of the receiver operating characteristic (ROC) curve for each class is shown. A typical ROC would have the x-axis plotted to 100%. We plot only up to 5%, to reduce the amount of overlap in the individual class plots along the *y*-axis and to improve clarity. Because the minimum specificity is 0.952, plotting up to 5% is a sufficient maximum for the x-axis. CSK, cytoskeleton; CYT, cytoplasm; END, endoplasmic reticulum; EXC, extracellular; GOL, golgi; LYS, lysosome; MIT, mitochondria; NUC, nucleus; PLA, plasma membrane; POX, perixosome.

An ROC curve depicts the relationship between specificity and sensitivity for a single class. The ROC curve for the perfect classifier would result in a straight line up to the top left corner, and then straight to the top right corner, indicating that a single score threshold can be chosen to separate all of the positive examples of a class from all of the negative examples. Figure 2 shows the ROC curve for each class in ngLOC. Each point in the curve is plotted based on different confidence score (CS) thresholds. For all classes except CYT and NUC, the ROC curves remain very close to the left side of the chart, primarily because the majority of classes have very high specificity at all CS thresholds. This is a desirable characteristic of ROC curves. Although PLA and mitochondria (MIT) have a high rate of false positives at the lowest score thresholds, the rate of true positives remains high, indicating that a good discriminating threshold exists for these classes. CYT has a high rate of false positives for lower score thresholds, again confirming that CYT is a class that is prone to over-prediction. This is also confirmed by its low precision (0.828). The other class that is prone to over-prediction is NUC, exhibiting the lowest precision of all 10 classes (0.807). NUC has the lowest specificity as well. This is probably a result of the characteristics of the short nuclear localization signals (NLSs) that exist on nuclear proteins. These NLSs can vary significantly between species. The ngLOC method, which uses a 7-gram peptide to explore the protein sample space along the entire length of the protein, is probably discovering many of these NLSs in the nuclear sequences. Because the dataset contains many examples of nuclear proteins among many species, many candidate NLSs will be discovered, thereby leading to over-prediction of nuclear proteins.

To obtain the sensitivity for multi-localized sequences, we consider two types of true positive measures: at least one of the two localizations had the highest probability, and both localizations had the top two probabilities. The overall accuracy of at least one localization being correctly predicted was 81.88%, and for both localizations being correctly predicted it was 59.7%. When considering the accuracy of both localizations being predicted to be within the top three most probable classes, the accuracy increased to 73.8%, suggesting that this method is useful in predicting multi-localized sequences.

### Evaluation of the confidence score

A probabilistic confidence measure is an important part of any predictive tool, because it puts a measure of credibility on the output of the classifier. Table 2 demonstrates the utility of our CS (range: 0 to 100) in judging the final prediction for each sequence. We found that a score of 90 or better was attributed to 37.5% of the dataset, with an overall accuracy of 99.8% in this range. About 86% of the dataset had a CS of 30 or higher. Although the accuracy of sequences scoring in the 30 to 40 range was only 70.1%, the cumulative accuracy of all sequences scoring 30 or higher was 96.2%. We found that the overall accuracy of the classifier proportionally scaled very well across the entire range of CSs.

**Table 2:** Benchmarking the performance of ngLOC (7-gram) against its confidence score

	Confidence score									
	
	0	10	20	30	40	50	60	70	80	90
% of dataset	0.0	2.4	11.8	6.1	4.4	4.5	5.8	9.3	18.1	37.5
% overall accuracy	0.0	56.2	41.4	70.1	88.3	93.0	97.0	98.1	99.2	99.8
Cumulative % of data:	100.0	100.0	97.6	85.7	79.6	75.2	70.7	64.9	55.6	37.5
Cumulative % overall accuracy	88.8	88.8	89.6	96.2	98.3	98.8	99.2	99.4	99.6	99.8

This table shows how the confidence score associated with each prediction relates to the overall accuracy. The higher the score, the more likely the prediction is to be the correct one. For example, all sequences scoring 90 or better had an accuracy of 99.8%. About 80% of the dataset was scored 40 or higher with a cumulative accuracy of 98.3%.

In Table 2, we present the performance of ngLOC under the restriction that the correct localization for a given sequence was predicted as the top most probable class. To understand how close ngLOC was on misclassifications, we expanded our true positive measure by considering correct predictions within the top four most probable classes. As shown in Table 3, for single-localized sequences, the overall accuracy jumped from 88.8% to 94.5% when the correct prediction is considered within the top three most probable classes. Although this improved accuracy has no meaning for single-localized sequences, it indicates that the majority of misclassifications were missed by a narrow margin. For multi-localized sequences the classifier predicted both correct localizations as the top two most probable classes 59.7% of the time; however, the classifier predicted both correct localizations within the top three or four classes with accuracies of 73.8% and 83.2%, respectively. We also considered the accuracy of only those sequences localized into both the cytoplasm (CYT) and nucleus (NUC), because they represent 51.6% of our set of sequences with two localizations. As expected, the accuracy increased, with at least one correct localization predicted within the top three with an accuracy of 99.5%, and both localizations predicted at an accuracy of 96.3% in the top four most probable classes. The high performance for sequences localized to both CYT and NUC is partly attributed to the fact that this combination of organelles has the largest representation of all multi-localized sequences in the dataset (1,120 out of 2,169).

**Table 3:** Rank of correct class single-localized and multi-localized sequences using a 7-gram model

	Rank of correct class			
	
	1	2	3	4
Single-localized only	88.8^a^	92.2	94.5	96.3
CYT-NUC: 1 correct	88.2^a^	96.1	99.5	100.0
CYT-NUC: both correct		66.5^a^	82.9	96.3
All multi-localized: 1 correct	81.9^a^	92.0	96.1	97.4
All multi-localized: both correct		59.7^a^	73.8	83.2

This table shows the percent of the data that had the correct localization predicted within the top *r *most probable classes, where *r *is the rank of the correct class. ^a^Items representing the overall accuracy of ngLOC on those sequences specified. CYT, cytoplasm; NUC, nuclear.

### Evaluation of the multi-localized confidence score

It is known that a significant number of sequences in eukaryotic proteomes are localized to multiple subcellular locations; a predominant fraction of such sequences shuttle between or localize to both the cytoplasm and nucleus. To differentiate single-localized sequences from those that are multi-localized, we developed a multi-localized confidence score (MLCS). We evaluated the MLCS on the entire dataset, and considered the accuracy on multi-localized sequences over different MLCS thresholds. For accuracy assessment in this test, a prediction is considered to be a true positive if both correct localizations are the top two most probable classes, which is the most stringent requirement possible. As shown in Table 4, 76% of the multi-localized sequences scored an MLCS of 40 or higher, whereas 81% of the single-localized sequences have MLCS scores under 40. Over 20% of multi-localized sequences received a score of 90 or better, as compared with only 0.2% of single-localized sequences in this range. Multi-localized sequences in this range had both localizations correctly predicted 98.7% of the time. These results are very promising, considering that multi-localized sequences comprise less than 10% of our entire dataset. In general, the higher the MLCS, the more likely the sequence is not only to be multi-localized but also to have both correct classes as the top two predictions. Table 5 shows examples of the MLCSs and CSs output by ngLOC for a few multi-localized sequences.

**Table 4:** Evaluation of MLCS against single-localized and multi-localized sequences

	MLCS									
	
	0	10	20	30	40	50	60	70	80	90
% of Single-localized data	25.9	21.2	12.6	21.1	13.6	3.1	1.2	0.6	0.4	0.2
Cumulative %, single-localized data	100.0	74.1	52.9	40.3	19.2	5.6	2.4	1.2	0.6	0.2
% of Multi-localized data	1.7	2.1	2.3	17.9	26.2	7.8	6.2	5.3	10.0	20.5
% Overall accuracy, multi-localized sequences only	36.1	45.7	46.9	20.3	34.5	63.3	83.7	86.2	94.4	98.7
Cumulative %, multi-localized data	100.0	98.3	96.2	94.0	76.0	49.8	42.0	35.8	30.5	20.5
Cumulative % accuracy, multi-localized sequences only	59.7	60.1	60.4	60.7	70.3	89.1	93.9	95.6	97.3	98.7

This table shows the percentage of the dataset that resulted in different ranges of the MLCS, as well as the overall accuracy and cumulative accuracy of multi-localized sequences in that range. MLCS, multi-localized confidence score.

**Table 5:** Examples of prediction for multi-localized sequences

Name	Correct	MLCS	CYT	END	GOL	CSK	LYS	MIT	NUC	PLA	EXC	POX
TAU_MACMU	CYT/PLA	98.2	49.1^a^	0.2	0.1	0.1	0.0	0.3	0.6	49.2^a^	0.3	0.1
CTNB1_MOUSE	CYT/NUC	85.1	49.8^a^	0.1	0.0	0.0	0.0	0.1	42.2^a^	7.5	0.2	0.0
3BHS2_RAT	END/MIT	97.9	0.4	48.9^a^	0.2	0.1	0.0	49.1^a^	0.3	0.4	0.4	0.1
SIA4A_CHICK	GOL/EXC	85.0	2.4	1.8	42.4^a^	0.6	0.0	1.8	2.5	4.6	43.7^a^	0.2
GGH_HUMAN	LYS/EXC	69.1	4.4	3.1	2.1	2.0	33.7^a^	3.2	5.9	5.4	39.9^a^	0.3

This table presents examples of multi-localized sequences predicted with a high multi-localized confidence score (MLCS) value. The 'name' column represents Swiss-Prot entry names. The 'correct' column shows both organelles in which the sequence is localized into. The remaining columns show the confidence score for each possible localization. CSK, cytoskeleton; CYT, cytoplasm; END, endoplasmic reticulum; EXC, extracellular; GOL, golgi; LYS, lysosome; MIT, mitochondria; NUC, nucleus; PLA, plasma membrane; POX, perixosome. ^a^These indicate the two correct localizations for each sequence.

### Comparing ngLOC with other methods

We evaluated the performance of ngLOC by comparing it with that of existing methods. Comparisons were made in three ways: by using the ngLOC dataset to train and test other methods; by testing ngLOC on another dataset; and by training and testing ngLOC on another dataset.

**Figure 3:**
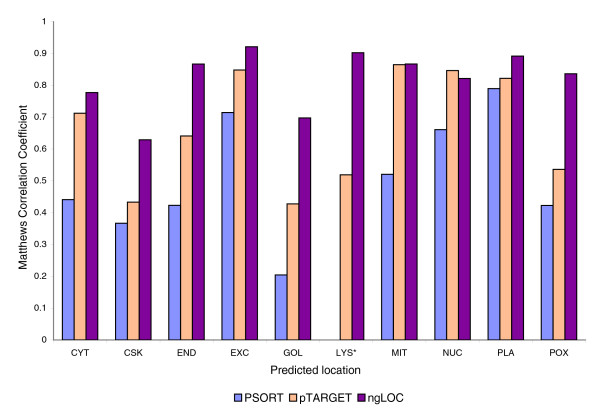
Comparison of predictions from three methods on the ngLOC dataset. Three methods, PSORT, pTARGET, and ngLOC, were evaluated by comparing the Matthews Correlation Coefficient (MCC) for each localization. The MCC was chosen because it provides a balanced measure between sensitivity and specificity for each class [23]. *The LYS location was omitted from PSORT predictions because PSORT predicts this class as part of the vesicular secretory pathway. CSK, cytoskeleton; CYT, cytoplasm; END, endoplasmic reticulum; EXC, extracellular; GOL, golgi; LYS, lysosome; MIT, mitochondria; NUC, nucleus; PLA, plasma membrane; POX, perixosome.

For our first test, we compared ngLOC against two existing methods, namely PSORT [24] and pTARGET [11]. Both of these methods are widely used by the research community, can predict 10 or more subcellular locations, and are freely available for offline analysis. For uniformity, we used a random selection of 80% of our dataset for training and 20% for testing. The overall accuracies of PSORT, pTARGET, and ngLOC are 72%, 83%, and 89%, respectively. We chose to compare these three methods using the MCC values as the comparative measure, because it is the most balanced measure of performance for classification. Figure 3 compares the MCC values on each of the 10 classes for all three methods. Our method showed a respectable improvement across all locations over PSORT and pTARGET, with the exception of pTARGET's accuracy on NUC, which had a slightly higher MCC than did ngLOC. In particular, ngLOC exhibited a significant improvement in all of the classes that had the smallest representation in the dataset (cytoskeleton [CSK], endoplasmic reticulum [END], golgi apparatus [GOL], lysosome [LYS], and perixosome [POX]), which are typically difficult to predict.

For our next comparative test, we found a similar dataset that has been used by the research community, namely PLOC (Protein LOCalization prediction) [8]. The primary differences between our data and PLOC's are in the version of the Swiss-Prot repository from which the sequences were acquired, the level of sequence identity assumed in the dataset, and the multi-localized annotation in our dataset. Sequences with up to 80% identity were allowed in the PLOC dataset, whereas all sequences with less than 100% identity were allowed in the ngLOC dataset. We disregarded sequences from the PLOC dataset that are localized into the chloroplast and vacuole, because we do not consider plant sequences. We built both a 6-gram and a 7-gram model using our entire dataset, and used the PLOC dataset for testing purposes. We had overall accuracies of 88.04% and 85.64%, respectively, both of which compared favorably with the 78.2% overall accuracy reported by PLOC. It is important to note that the optimal value of *n *in ngLOC is dependent on the amount of redundancy in the data being tested. A 6-gram model performed better than a 7-gram one, which confirms the lower redundancy in the PLOC dataset than in the ngLOC dataset. We observed that there were some predictions with a CS of 90 or greater but were misclassified by ngLOC. We discovered that all sequences predicted with this level of confidence that were misclassified by ngLOC were due to incorrect annotation, probably because of the PLOC dataset being outdated (see Additional data file 1 [Supplementary Table 1] for some examples). Each one was verified in the latest Swiss-Prot entry as matching our prediction. We also found instances in which some of the predictions misclassified by ngLOC were actually multi-localized and should have been considered correct as well (Additional data file 1 [Supplementary Table 2]. Our performance results are without correcting any annotations in the PLOC dataset. We believe that updated annotations in the PLOC dataset, as well as updates that label multi-localized sequences, would further improve the accuracy of ngLOC on the PLOC dataset.

For our final comparative test, we modified ngLOC to predict 12 distinct classes, and used the complete PLOC dataset (with original annotations and all 12 localizations) for both training and testing on our method, using a 10-fold cross-validation for performance analysis. On a 6-gram model, the overall accuracy was 82.6%, which again compared favorably with PLOC's accuracy of 78.2%. We found numerous misclassifications that had a correct second-highest prediction (see Additional data file 1 [Supplementary Table 3] for example predictions). In fact, out of 12 possible classifications, ngLOC predicted the correct localization to be within the top two most probable classes 88.7% of the time. It is interesting to note that even in this test we discovered some sequences that were misclassified according to PLOC annotations, but the prediction by ngLOC was consistent with the latest release of Swiss-Prot (Swiss-Prot:P40541 and Swiss-Prot:P33287). We also discovered instances where the sequence is multi-localized, and ngLOC predicted the location that was not annotated in the PLOC dataset (for instance, Swiss-Prot:P40630 and Swiss-Prot:P42859]. Nevertheless, we believe that these annotations were correct at the time the PLOC dataset was constructed. These results underscore the robustness of our method and usefulness of its CS, because we were able to identify outdated annotations in the PLOC dataset, identify potential multi-localized proteins in data not annotated accordingly, and consider alternate localizations beside the predicted class when the CS is low, suggested by the high accuracy when considering the top two classifications.

### Evaluating ngLOC-X for proteome-wide predictions

We extended the core ngLOC method to allow classification of proteins from a single species. We call this method ngLOC-X, which is based on the model depicted in equation 9 (see Materials and methods, below). Assessing the performance of ngLOC-X proved challenging, because only a small percentage of each proteome has subcellular localizations annotated by experimental means, and therefore it is impossible to infer an exact accuracy measurement on proteome-wide predictions. However, subsets of these proteomes are represented in the ngLOC dataset, and so performance analysis can be inferred from these subsets. We chose two species for performing extensive analysis: mouse (3,596 represented sequences out of 23,744) and fruitfly (753 represented sequences out of 9,997). (Human had the largest set, with 5,945 represented sequences; we did not test this subset because of the amount of data that would need to be removed from the core ngLOC dataset.) For each species, we extracted the represented protein sequences from the ngLOC dataset and trained ngLOC on the remaining data. After training, we ran a 10-fold cross-validation on the extracted data, comparing the performance results between the standard ngLOC model against ngLOC-X. For this test, we examined the predictions of only single-localized sequences, resulting in 3,214 sequences from mouse and 683 sequences from fruitfly for analysis.

The standard ngLOC model achieved overall accuracies of 93.5% and 79.5% for mouse and fruitfly, respectively. For ngLOC-X, the overall accuracy stayed the same for mouse, and increased to 80.5% for fruitfly. The average sensitivity (often reported as normalized overall accuracy) improved as well, increasing from 86.9% to 87.5% in mouse, and from 72.6% to 74.0% in fruitfly. Although the gains in overall accuracy and sensitivity are not significant, we noted a significant increase in the number of sequences predicted with high confidence. For mouse, ngLOC predicted 39.1% of the data with a CS above 90 at 99.8% accuracy, whereas ngLOC-X predicted 52.9% of the data in the same range at the same accuracy. Fruitfly exhibited the same effect, with ngLOC predicting 28.1% of the data with a CS above 70 at 99.0% accuracy, whereas ngLOC-X predicted 38.7% of the data in the same range at 99.2% accuracy. We are sure that this is an artifact of adjusting the *n*-gram probabilities to reflect the proteome being predicted. Nevertheless, this test showed us that incorporating the proteome for species X in the model, as required for ngLOC-X, did not have a negative effect on the performance compared with the standard ngLOC model, while improving the coverage of the proteome predicted with high confidence.

We sought to determine how the predictions would be affected when ngLOC-X was trained on the proteome of one species, and tested on a different species. When testing the mouse sequences on ngLOC-X trained for fruitfly, the overall accuracy and normalized accuracy again stayed the same. However, when testing fruitfly on ngLOC-X trained for mouse, the overall accuracy dropped from 80.5% to 79.2%, which was slightly worse than the standard ngLOC model. These tests showed us that a species with high representation in the training data will not result in any improvement in overall accuracy by tuning the model for a specific proteome, but that a species with low representation will yield the greatest benefit when the model parameters are tuned specifically for that species.

Our next test was to examine the instances in these proteome subsets in which ngLOC and ngLOC-X generated different predictions. For the mouse data, we found 62 sequences out of the 3,214 single-localized sequences predicted that resulted in different predictions between the two methods. The standard ngLOC method had 15 of these sequences predicted correctly, whereas ngLOC-X had 16. For the fruitfly predictions, there were 38 sequences out of the 683 sequences with different predictions. Of these, ngLOC had 10 instances that were predicted correctly, whereas ngLOC-X had 17 correct predictions.

Although most of these improvements demonstrated by ngLOC-X are statistically insignificant, fruitfly exhibited a relatively greater improvement from the ngLOC-X method than did mouse. We also discovered in both cases that almost all sequences with different predictions between the two methods were instances predicted with a low CS (for example, a CS value <40.) These results may be explained by recognizing that low-confidence predictions are more likely for sequences from a species that does not have a high representation of an evolutionarily close species in the training data. The ngLOC dataset has a higher number of proteins from species closely related to mouse (the mammalian proteins) than to fruitfly. This is confirmed by the overall accuracies reported from ngLOC for mouse and fruitfly, which were 93.5% and 79.5%, respectively; it is also confirmed by the fact that 90.8% of the mouse data were predicted with a CS of 40 or greater, whereas fruitfly only had 66.6% of the data predicted in the same CS range. Moreover, we believe that ngLOC-X will have the most benefit on the predictions from a species with low representation in the training data. This is confirmed by the following observations. First, there was a noticeable increase in the overall and normalized accuracy between ngLOC and ngLOC-X on fruitfly, whereas mouse did not benefit from ngLOC-X. Second, our cross-species test showed that testing mouse predictions on ngLOC-X trained for fruitfly did not affect the accuracy, whereas fruitfly showed slightly worse performance than the standard ngLOC method when tested on ngLOC-X trained for a mouse. Based on these findings, it is evident that ngLOC-X will show improvement in the accuracy of low-confidence predictions over ngLOC. If the sequences from a species being predicted have a high representation of evolutionarily closer species in the training data (such as mouse), then ngLOC-X has little value in final predictive accuracy. In either case, ngLOC-X never resulted in a decrease in performance compared with ngLOC, and resulted a significant increase in high confidence predictions; hence, it is the method of choice for proteome-wide prediction of subcellular localizations.

Our final test was to compare location-wise predictions between ngLOC and ngLOC-X on the entire proteome for mouse and fruitfly. For this test, we trained both methods using the entire ngLOC dataset, and then applied each method on the entire Gene Ontology (GO)-annotated proteome data obtained. Table 6 shows the percentage of sequences localized into each possible class. The prediction for each sequence is determined by observing the most probable class predicted, and assigning that class as the prediction. In this test, all predictions are considered, meaning that no CS threshold is assumed, and neither are multi-localized sequences determined. Mouse had 56.8% of the 23,744 predictions for ngLOC generated with a CS of 40 or greater, as compared with 58.1% for ngLOC-X. Fruitfly had 26.3% of the 9,997 predictions for ngLOC generated in the same range, as compared with 35% for ngLOC-X. Again, we observed a more substantial increase in coverage for ngLOC-X in the predictions for the fruitfly proteome, a species with low representation, whereas mouse showed little increase in coverage for the same range. There were 2,555 out of 23,744 (10.76%) different predictions between ngLOC and ngLOC-X for mouse, and 1,126 out of 9,997 (12.02%) different predictions for fruitfly. This test showed us that when considering predictions on a proteome level, even a highly represented species such as mouse will result in many predictions of low confidence, and thus can potentially benefit from ngLOC-X as well.

**Table 6:** Comparison of location-wise prediction percentages for mouse and fruitfly

	Mouse (*M. musculus*)		Fruitfly (*D. melanogaster*)	
	
Location	ngLOC	ngLOC-X	ngLOC	ngLOC-X
% CYT	15.86	16.32	13.35	14.60
% CSK	0.88	2.10	0.37	1.29
% END	2.36	3.37	1.76	3.04
% EXC	11.6	12.26	12.50	13.10
% GOL	1.27	2.09	0.97	1.60
% LYS	0.46	0.98	0.24	0.67
% MIT	3.07	4.77	3.46	5.37
% NUC	33.22	30.13	43.90	39.17
% PLA	30.93	27.42	23.23	20.71
% POX	0.33	0.58	0.21	0.44

CSK, cytoskeleton; CYT, cytoplasm; END, endoplasmic reticulum; EXC, extracellular; GOL, golgi; LYS, lysosome; MIT, mitochondria; NUC, nucleus; PLA, plasma membrane; POX, perixosome.

We can only offer educated speculation regarding the results, because accurate annotation is not available. However, the proteome-wide predictions obtained by ngLOC-X are closer to what we expect than those obtained by ngLOC. For example, in our previous work, in which we used a completely different method [16], we estimated that 6.3% of the proteome of the fruitfly and 4.6% of the proteome of the mouse is localized in the mitochondria. Our 5.4% and 4.8% predicted with ngLOC-X for fruitfly and mouse, respectively, compared favorably with our former results, and showed significant improvement for mitochondrial estimates over ngLOC in both cases. All of our comparative tests of ngLOC versus ngLOC-X showed that ngLOC-X was a valuable addition to the core ngLOC method.

### Estimation of subcellular proteomes of eight eukaryotic species

We have used ngLOC-X to estimate the subcellular proteomes of eight different eukaryotic species. With the exception of yeast, proteomes of eukaryotic model organisms have a significant portion of hypothetical proteins (about 25% to 40%). To avoid predictions on hypothetical proteins, we generate predictions on a subset of the proteome containing at least one annotated GO concept, namely those proteins that have been experimentally validated or closely related to proteins with experimental validation at some level. We then use these predictions to generate estimates of the subcellular proteome for each species.

To generate the complete results, we trained ngLOC-X using the entire ngLOC dataset. Predictions were generated for the GO-annotated subset of sequences for each proteome. We selected a CS threshold that allows inclusion of all predictions except those of very low confidence. One reason why we did this was that ngLOC predicts only 10 subcellular locations. However, there are other relatively minor organelles in eukaryotic cells that proteins may localize into. (For example, ngLOC does not predict sequences targeted for the vacuole. Although this organelle is nearly nonexistent in higher eukaryotic cells, it is significant in yeast cells.) These sequences will probably result in a very low CS, because they have no representation in the training data. The other reason why we selected a CS threshold was that sequences that have a low homology measure with respect to any other sequence in the ngLOC training data will be hard to classify, and will also result in a low CS. For these two reasons, we chose a CS threshold (CSthresh) of 15 as the cutoff value to aid in eliminating these sequences from the proteome estimation. With this threshold, ngLOC covered an impressive range of 94.52% to 99.82% of the tested proteomes (Table 7). The proteome estimations are based on the percentage of sequences predicted with a CS of greater than or equal to CSthresh. We chose an MLCS threshold (MLCSthresh) of 60 to estimate the percentage of the proteome that is multi-localized. According to Table 4, in a tenfold cross validation test, 42% of the multi-localized sequences in ngLOC were predicted with an MLCS of greater than or equal to 60 at an accuracy of 93.9%, whereas only 2.4% of single-localized sequences were incorrectly predicted as multi-localized at this threshold. This is a conservative threshold chosen to emphasize higher accuracy on multi-localized sequences without over-prediction. We also report the percentage of the proteome multi-localized into both the cytoplasm (CYT) and nucleus (NUC), because more than half of the multi-localized sequences in the ngLOC training dataset are localized between these two organelles. Table 7 shows the complete results. (See Additional data file 1 [Supplementary Table 4] for the corresponding chart containing numeric estimates of the fractions in Table 7.)

**Table 7:** Estimation of the subcellular proteomes of eight eukaryotic organisms

	Yeast (*S. cerevisiae*)	Worm (*C. elegans*)	Fruitfly (*D. melano*)	Mosquito (*A. gambiae*)	Zebrafish (*D. rerio*)	Chicken (*G. gallus*)	Mouse (*M. musculus*)	Human (*H. sapiens*)	Range
Proteome	5,799	22,400	13,649	15,145	13,803	5,394	33,043	38,149	
GO annotated	5,486	12,357	9,997	8,847	10,106	4,363	23,744	24,638	
% ngLOC coverage	97.48	94.92	96.73	97.94	98.64	9,9.82	94.79	94.52	94.79-99.82
Proteome estimated	5,653	21,262	13,203	14,833	13,616	5,384	31,320	36,059	

% CYT	15.22	14.80	12.74	14.43	15.01	13.66	13.44	14.14	12.74-15.22
% CSK	1.07	1.19	1.05	1.11	1.31	1.24	1.50	1.48	1.05-1.50
% END	2.71	3.47	2.85	3.25	3.34	2.53	2.99	3.04	2.53-3.47
% EXC	8.88	12.60	12.26	14.28	9.91	12.65	11.52	11.71	8.88-14.28
% GOL	1.48	1.31	1.40	1.07	1.68	1.47	1.52	1.56	1.07-1.68
% LYS	0.11	0.58	0.55	0.53	0.65	0.44	0.59	0.67	0.11-0.67
% MIT	9.55	5.84	4.86	5.52	4.72	4.16	4.24	4.80	4.16-9.55
% NUC	33.53	29.75	37.38	29.50	30.31	28.24	27.35	28.38	27.35-37.38
% PLA	16.19	24.41	20.06	21.36	21.66	22.78	27.18	24.08	16.19-27.18
% POX	0.54	0.66	0.42	0.48	0.51	0.25	0.44	0.46	0.25-0.66

% Single-localized	89.29	94.60	93.59	91.53	89.11	87.42	90.77	90.32	
% Multi-localized	10.71	5.40	6.41	8.47	10.89	12.58	9.23	9.68	
% CYT-NUC	6.49	2.36	2.76	3.44	5.40	6.27	4.51	4.74	

This chart presents the location-wise percentages of the proteome predicted to localize into one organelle. (For example, 9.55% of the yeast proteome is localized to the mitochrondria only.) These percentages sum to the total size of the proteome estimated to be single-localized. We also present the estimated percentage of the proteome that is localized to multiple organelles. The percentage of the proteome estimated to localize to both the cytoplasm and nucleus is also displayed. The coverage is determined with a confidence score (CS) threshold of 15. Multi-localized sequences are determined with a multi-localized confidence score (MLCS) threshold of 60. CSK, cytoskeleton; CYT, cytoplasm; END, endoplasmic reticulum; EXC, extracellular; GO, Gene Ontology; GOL, golgi; LYS, lysosome; MIT, mitochondria; NUC, nucleus; PLA, plasma membrane; POX, perixosome.

Overall, the fractions of subcellular proteomes scaled consistently across the different species, as shown in the last column of Table 7. We observed that the percentage of sequences localized into the endoplasmic reticulum (END), golgi apparatus (GOL), and perixosome (POX) tend to remain relatively consistent across species, with average percentages of 3.0%, 1.44%, and 0.5%, respectively. In contrast, the fractions of the subcellular proteomes with relatively large percentages (cytoplasm [CYT], mitochondria [MIT], nuclear [NUC], plasma membrane [PLA], and extracellular [EXC]) varied widely across different species. This variation is expected, because as multicellular eukaryotes evolved with higher complexity, consolidation of specific cellular functions to defined organelles took place, resulting in the sequestering of corresponding proteins to these organelles. As a result, more variation is observed in the proteome sizes of larger organelles. Nevertheless, the fraction of subcellular proteomes reported for mouse and human are very similar, which is expected because of their close evolutionary distance. The size of the yeast mitochondrial proteome estimate in this study (9.55%) agrees with those previously reported (about 10%) by computational methods [9,16], and closely matches the experimental estimates reported (13%) [25]. Similarly, about 1,500 nucleus-encoded mitochondrial proteins have been estimated in the human mitochondria [4,26] and our estimate of 4.8% corresponds to 1,730 proteins (Table 7 and Additional data file 1 [Supplementary Table 4] contain numeric proteome estimates), suggesting that ngLOC-X estimates are on par with those obtained by other computational and experimental approaches.

Some of the organelles indicate a trend related to the evolutionary complexity of the species being predicted. The fraction of proteomes localized to the cytoskeleton (CSK) and golgi (GOL) appear to exhibit an increasing trend with the evolutionary complexity of the species, whereas mitochrondria (MIT) and nucleus (NUC) indicate a slight decreasing trend. For the other organelles, such trends are not noticeable. Nevertheless, we should like to point out that the proteomes compared in this study are not evolutionarily equidistant, which makes it difficult to infer trends in the evolution of organellar proteomes.

Table 8 shows the prediction percentages for all single-localized and multi-localized sequences in the human proteome. The boxed areas in the table represent the percentages of single-localized data, as presented in Table 7. The remaining areas in the table represent multi-localized percentages. The sum of the nonboxed cells in Table 8 will result in the percentage multi-localized value in Table 7. Although sequences localized to both the cytoplasm and nucleus occupy a significant portion of the multi-localized subcellular proteome, we found that approximately one-third of the sequences localized into the cytoplasm were predicted to localize into other organelles as well. This is probably because the cytoplasm is the default location for protein synthesis as well as the hub of cellular core metabolism. Similarly, almost 1% of the proteome consisted of secreted proteins that were also localized to the plasma membrane.

**Table 8:** A matrix showing estimated fractions of subcellular proteomes on the human proteome

Location	CYT	CSK	END	EXC	GOL	LYS	MIT	NUC	PLA	POX
CYT	14.14^a^									
CSK	0.64	1.48^a^	0.01							
END	0.10		3.04^a^							
EXC	0.22	0.01	0.04	11.71^a^						
GOL	0.29	0.03	0.31	0.17	1.56^a^					
LYS	0.02			0.03	< 0.01	0.67^a^				
MIT	0.31		0.07	0.02	< 0.01		4.80^a^			
NUC	4.74	0.07	0.09	0.12	0.01		0.09	28.38^a^		
PLA	0.77	0.02	0.14	0.94	0.09	0.00	0.03	0.19	24.08^a^	
POX	0.05			< 0.01			0.03			0.46^a^

This chart shows the percentages of the proteome estimated to localize over 10 different organelles. ^a^These cells represent the percentage of sequences predicted to single-localize; all other cells represent multi-localized sequences. The values are based on a *CSthresh *of 15 and *MLCSthresh *of 60. CSK, cytoskeleton; CYT, cytoplasm; END, endoplasmic reticulum; EXC, extracellular; GOL, golgi; LYS, lysosome; MIT, mitochondria; NUC, nucleus; PLA, plasma membrane; POX, perixosome.

We compared our estimates with those generated using the PSLT method [21]. Our estimates of the human subcellular proteome largely agree with those reported by PSLT, with the exception of a difference in the number of multi-localized sequences (16.0% versus 9.7% reported by ngLOC-X), which is probably due to our conservative choice for MLCSthresh (≥60). (For comparison, an MLCSthresh ≥50 resulted in 13.4% of the predictions being multi-localized.) We also show a significant difference for those proteins targeted for the plasma membrane (17.1% versus 24.1% reported by ngLOC-X). This may be significant, because our predictions are based on 24,638 sequences from the human proteome, as compared with PSLT's predictions on 9,793 sequences. Moreover, proteins localized to the plasma membrane have large coverage in the ngLOC dataset. These reasons suggest that our estimates are certainly plausible. Additional data file 1 (Supplementary Tables 6 to 21) provides the complete prediction matrices generated for all eight eukaryotic species.

### Biological significance of discriminatory *n*-grams

It is well known that functional domain regions of proteins are highly conserved because they define a vital part of the overall functionality of the protein. From our previous studies, we observed that about 74% of the functional domains are localized exclusively to only one of the 10 subcellular locations [11]. Hence, we wondered whether we could observe any relationship between discriminatory *n*-grams and their occurrence in the domain regions in a protein. To perform this test, we downloaded domain definitions from the InterPro database [27]. Only domains definitions that were at least as long as the *n*-gram length used in the ngLOC model were considered. We mapped these domain definitions onto the single-localized proteins in the ngLOC dataset. This resulted in 15,109 protein sequences that had some portion of its sequence mapped to a functional domain. Overall, 75.5% of the *n*-grams in these sequences were mapped to a domain. (We say that an *n*-gram is mapped to a domain only if the entire *n*-gram falls within the bounds of a domain.) Different localization classes had different coverage of *n*-grams in domain regions, ranging from 53.7% (nuclear [NUC]) to 86.8% (lysosome [LYS]). Additional data file 1 (Supplementary Table 5) provides the complete results of this analysis.

In this study, an *n*-gram is said to be highly discriminatory if its occurrence in a protein sequence is highly correlated with a specific localization. We consider a very conservative, strict definition of a discriminatory *n*-gram as an *n*-gram that occurs at least five times over all sequences but in only one localization class in the ngLOC dataset. Based on this definition, we found that only 15.0% of all *n*-grams were highly-discriminatory. However, 91.4% of all discriminatory *n*-grams occurred entirely within a domain region, suggesting that the discriminatory *n*-grams indeed originate from the domain regions. It should be noted that the number of discriminatory *n*-grams found in domain regions vary among different subcellular classes (ranging from 80.2% to 97.5%). Nevertheless, all of these occurrences are statistically significant compared with their expected values, as shown in Additional data file 1 (Supplementary Table 5).

## Discussion

### ngLOC method development

A multinomial naïve Bayes model is a simplistic yet effective model when used in conjunction with the *n*-gram model for representing proteins. The *n*-gram model is able to capture sequence homology while allowing for differences due to insertion, deletion, or mutation. This model effectively shrinks the protein sequence space, thereby allowing a higher degree of redundancy between proteins of different classes that could not be achieved by considering the entire protein sequence. It should be noted that the optimal value of *n *chosen is highly dependent on two factors: the number of sequences in the training data and the measure of sequence similarity in the training data. Generally, both large datasets and datasets with high sequence similarity will need longer *n*-grams for effective classification, although larger values of *n *will result in a model that overfits the training data. Additionally, this has an affect on the CS. If the dataset is large and highly similar, we found that short *n*-grams lead to probabilities that are all relatively close in value, which results in CSs that all fall within a very tight range. The reason for this is that the total number of *n*-grams in equation 4 is proportionally large with respect to the size of the dataset. For example, when using a 2-gram model on our dataset, the scores for the entire dataset all ranged between 8.41 and 11.72, but when we use a 7-gram model the range is 0.0 to 99.21. Although the scores were in a tight range for the 2-gram, we observed the same relationship between relative score value and overall accuracy. It would be easy to re-scale the scores for performance analysis to fall within similar ranges across all models.

From the protein feature space point of view, a different sized *n*-gram will map protein surface features differently. We believe that the high performance exhibited by 6- to 8-gram models (Figure 1) is due to the fact that these *n*-gram peptides are ideal for mapping the secondary structure space of proteins. Secondary structure elements are vital for attaining a proper fold of a protein, and consequently are vital for its function. Hence, these secondary structures are distinctly conserved across proteins with different functions and from different subcellular locations.

### Comparison with other methods

Many recent methods, including PLOC, were based on SVMs [8,28,29]. As successful as some of these models have been, we determined that SVMs were not suitable for our needs. First, we plan to explore the most discriminatory *n*-grams in proteins between different subcellular organelles. With ngLOC, it will be easy to extract *n*-grams of interest from the model, because the relation between each *n*-gram and the integer identifier generated for use by the classifier is symmetric. However, with SVM-based methods, the kernel in the SVM projects the features of the data to a higher dimensional space to increase the likelihood of making the data linearly separable. Although one might discover excellent SVM parameters for a particular classification problem, it will be difficult to understand how the translated feature space is discriminating between classes. Second, as we have illustrated, a probabilistic measure ought to be considered a crucial part of any predictive model. Therefore, we determined that a pure probabilistic model was desired. Deriving this measure is a difficult feat for SVMs because of their nonprobabilistic output. Any attempt to derive such a measure with SVMs can be done only by creating another layer of classification to simulate a probability measure from the output of the SVMs. The risk of the simulated probability distribution overfitting the data used to generate the distribution is a known artifact with these methods [30]. The pure probabilistic confidence measure derived directly from the probabilities calculated from a method such as ngLOC will have a more consistent, scalable probability measure.

### Estimation of subcellular proteomes

Our model, ngLOC, was enhanced to allow dynamic adjustment of the model parameters specific to a proteome being estimated. This model, termed ngLOC-X, is useful for predicting the subcellular localization of the proteome of any species. Our proteome prediction results showed that although a single model can be used on a variety of species, better results can be had if the model is tuned for a specific species being considered (Table 6). If a single model is being used across numerous species, it is very important to include a broad spectrum of data across all species. Unfortunately, this is not possible because of the imbalanced nature of protein sequence data in the public domain. Our model, ngLOC-X, essentially extends the core ngLOC model by introducing a bias toward a single species being predicted. The accuracy and coverage of our model across species will continue to burgeon as the proteomes of new eukaryotic species become available. The eukaryotic species selected in this study represent a broad spectrum in the eukaryotic superkingdom (not including plants). Despite this, corresponding fractions of each subcellular proteome fall within a reasonable range across species (Table 7). This suggests that the proteome size corresponding to the core functionality of an organelle remains unchanged across species, whereas the observed variation in size allows for functionalities required by specific species for their adaptation. This hypothesis can be tested by studying the organellar proteomes at the domain level, and we aim to continue this work in the future.

### Discriminatory *n*-grams and functional domains

It is known that targeting signals such as KDEL/HDEL (for endoplasmic reticulum) and SKL (for peroxisomes) play a distinct role in transporting a protein to its destination in the cell. Nevertheless, ngLOC does not require prior knowledge regarding such signal peptides, and neither does it explicitly consider such information in the prediction process. Despite this, ngLOC is able to perform better than methods that explicitly use such information because each discriminatory *n*-gram is analogous to such signals. To support this argument, we demonstrated that 91.4% of all discriminatory *n*-grams originate from the domain regions of proteins (Additional data file 1 [Supplementary Table 5], which define the core function of a protein. The observations suggest that ngLOC predictions are based on functionally significant regions (domains) of the protein sequences, which are represented by *n*-grams covering the entire sequence space. In contrast, methods that rely on target signals generally scan only the amino-terminal or carboxyl-terminal regions of protein sequences, where such signals are located. It is likely that if the targeting signals are shorter than the *n*-gram, then the discriminatory *n*-grams represent both the signal as well as its neighborhood (which is often very important for transport). Similarly, if protein transport requires motifs that are longer than the *n*-gram, such motifs would be represented by multiple and mostly contiguous *n*-grams. Therefore, ngLOC need not have prior knowledge of specific targeting signals, because it is likely that analogous signals (discriminatory *n*-grams) are inherently identified *de novo *and used in establishing the localization prediction of the protein. Because of this plasticity, the ngLOC method has the ability to perform well on a number of locations, and hence it is highly suitable for proteome-wide prediction of subcellular localization.

## Conclusion

In this new age of proteomics there is great need for computational methods that can classify newly discovered proteins using information derived only from the primary sequence. Methods that predict the subcellular localization of a protein are an important part of meeting this need. In our study we have developed the ngLOC method, a Bayesian classifier that can predict the subcellular localization of a protein with superior performance against other methods of similar scope.

Because ngLOC is a probabilistic method, we were able to generate an extremely useful probabilistic confidence score (CS) that places a measure of credibility on each prediction. We have shown how this measure was used to determine the most likely localization for new proteins and possible annotation errors on known proteins. From this score, we also were able to develop a confidence measure to aid in determining multi-localized proteins as well, which is an important need in this area, because a significant part of the proteome is known to localize into multiple compartments. These scores developed in this study are sound and useful for predicting sequences localized to both single or multiple locations with high accuracy.

We extended the core ngLOC method, called ngLOC-X, and showed how it improved coverage for proteome-wide predictions over a single species by performing a comparative analysis of the results from both methods. We applied ngLOC-X to estimate ten distinct subcellular proteomes for eight eukaryotic model organisms. To our knowledge, this study presents the first estimate of ten distinct organelles on eight eukaryotic species with our coverage.

As with most computational models, the accuracy of ngLOC is completely dependent on the quality and coverage of the dataset used to train the model. Although many methods are unable to use large datasets because of computational limitations, ngLOC does not have these limitations. Clearly, modern day proteomics will continue to produce increasing amounts of experimentally determined data. The simplicity of the ngLOC model will enable it to easily incorporate these new data as they become available, thereby increasing the accuracy and coverage of ngLOC in the future. ngLOC can play a significant role in this field, when used in conjunction with experimental methods, to help meet the needs of the research community.

## Materials and methods

### ngLOC dataset

The dataset used for this task is a set of protein sequences taken from the Swiss-Prot database, release 50.0 [31], which contains experimentally determined annotations on subcellular localization. We applied the following filters to obtain high-quality data for testing and training our program: only eukaryotic, nonplant sequences were considered; sequences with predicted or ambiguous localizations were removed; sequences shorter than 10 residues in length were removed; all redundant sequences were removed; and sequences known to localize in multiple locations were manually checked and sorted to avoid errors caused by automated keyword-based sorting. The final set of training data consisted of a set of 28,056 sequences. Of these, 2,169 sequences were annotated with two distinct subcellular localizations, of which 1,120 were localized to both cytoplasm and nucleus. Location-wise distribution of this dataset is shown in Table 9.

**Table 9:** Distribution of proteins over subcellular localizations

Organelle	Code	Number of sequences
Cytoplasm	CYT	2,884
Cytoskeleton	CSK	248
Endoplasmic Reticulum	END	939
Extracellular	EXC	7,536
Golgi apparatus	GOL	282
Lysosome	LYS	166
Mitochrondria	MIT	2,442
Nuclear	NUC	4,658
Plasma membrane	PLA	6,530
Perixosome	POX	202
2 localizations annotated		2,169

Total		28,056

### Proteome datasets

We downloaded the proteomes of eight eukaryotic model organisms from the Integr8 database [32], which include *Saccharomyces cerevisiae *(yeast), *Caenorhibditis elegans *(worm), *Drosophila melanogaster *(fruitfly), *Anopheles gambiae *(mosquito), *Danio rerio *(zebrafish), *Gallus gallus *(chicken), *Mus musculus *(mouse), and *Homo sapiens *(human). Because 25% to 40% of these proteomes are hypothetical proteins (with the exception of yeast), we separated the curated proteome subsets containing annotation for at least one of the three GO concepts, but not including those with GO evidence codes: ND (no biologic data available), RCA (reviewed computational analysis), and NAS (nontraceable author statement).

### Performance measurements

We report standard performance measures over each subcellular location class, denoted as *c*_*j*_, including the following: sensitivity (recall), which is the fraction of data in class *c*_*j *_that were correctly predicted; precision, which is the fraction of data predicted to be in class *c*_*j *_that were actually correct; specificity, which is the fraction of data not in class *c*_*j *_that were correctly predicted; false positive rate, which is the fraction of data not in class *c*_*j *_that were incorrectly predicted to be in class *c*_*j*_; and MCC. The latter provides a measure of performance for a single class being predicted, where it equals 1 for perfect predictions on that class, 0 for random assignments, and less than 0 if predictions are worse than random [23].

We also report overall accuracy, defined as the fraction of data that were classified correctly, as a comparative measure of the overall performance of the classifier. Finally, we show a ROC curve as a graphical means of measuring the performance for each class. (All of our formulas used to measure performance are detailed in Additional data file 1.) All performance measurements are based on a standard 10-fold cross validation unless otherwise stated.

### The *n*-gram model for protein representation

Letting S denote the feature space used to represent all proteins, we develop S and our predictive model in light of the significant work that has been accomplished in the field of document classification. Cheng and coworkers [33] showed that using document classification techniques on the primary sequence can achieve good results on classifying protein families. In a typical document classification model, S is constructed by considering all possible words that may appear throughout the entire set of documents. Here, we consider subsequences of a protein of fixed length *n *as the equivalent of words in a document. In literature, these protein subsequences have been commonly called *n*-grams, *n*-mers, *n*-peptides, or simply words or subsequences of length *n *[34,35]. We adopt the term *n*-gram. In protein classification tasks using the *n*-gram model, S is constructed by considering all possible *n*-grams.

Formally, we let Σ represent the set of all possible amino acids, and |Σ| = 20. Given a dataset of protein sequences D, let *d*_*i *_be a sequence in D having *k *residues in length, where *d*_*i *_= (*s*_1_*s*_2 _... *s*_*k*_) and each *s*_*i *_∈ Σ. In an *n*-gram model, the size of the feature space grows exponentially with *n*, because |S| = |Σ|^*n*^. To illustrate, an integer variable typically requires four bytes of memory. If such a variable was used to keep track of the frequency of each of the possible 5-grams, the model would require 4 bytes × 20^5 ^features = 12.8 MB of memory, a 6-gram model would require 256 MB, and a 7-gram requires 5.1 GB of memory. Fortunately, for large values of *n*, relatively few *n*-grams actually occur in nature because of the evolutionary selection process, which requires a delicate mixture of various amino acid combinations in a peptide to sustain a fold. A simple analysis on the entire National Center for Biotechnology Information nonredundant dataset (2.7 million protein sequences at the time of this analysis) showed that an *n*-gram length as small as *n *= 5 had examples that never occurred. Therefore, to allow *n*-gram models for *n *> 5, we take advantage of the sparse nature of higher order *n*-grams by developing a one-to-one mapping between unique *n*-grams and the set of integers to be used as indices, as needed. This requires memory allocation only for *n*-grams that occur in the training data, thereby allowing exploration of large values of *n*.

### Naïve Bayes classifiers and subcellular localization

Bayesian predictive models have been effectively used in a variety of classification problems, including both document and protein classification tasks [33,36-38]. We give a brief derivation of the model in the context of protein subcellular localization prediction, using similar notation as depicted by McCallum and Nigam [36].

Given a protein sequence *d*_*i*_, a probabilistic approach to subcellular localization prediction is to develop a model to estimate the probability that *d*_*i *_is localized into each *c*_*j *_∈ C, where C represents the set of all possible localization classes. The classifier *h *predicts the localization of *d*_*i *_to the class that has the greatest posterior probability. Equation 1 shows this in probabilistic terms, and shows how the well known Bayes rule is used to derive an estimate for this probability.

h(di)=arg⁡max⁡cjP(cj|di)=arg⁡max⁡cjP(di|cj)P(cj)P(di)
 MathType@MTEF@5@5@+=feaafiart1ev1aaatCvAUfeBSjuyZL2yd9gzLbvyNv2Caerbhv2BYDwAHbqedmvETj2BSbqee0evGueE0jxyaibaiKI8=vI8tuQ8FMI8Gi=hEeeu0xXdbba9frFj0=OqFfea0dXdd9vqai=hGuQ8kuc9pgc9s8qqaq=dirpe0xb9q8qiLsFr0=vr0=vr0dc8meaabaqaciGacaGaaeqabaqadeqadaaakeaacaWGObGaaiikaiaadsgadaWgaaWcbaGaamyAaaqabaGccaGGPaGaeyypa0ZaaCbeaeaaciGGHbGaaiOCaiaacEgaciGGTbGaaiyyaiaacIhaaSqaaiaadogadaWgaaadbaGaamOAaaqabaaaleqaaOGaamiuaiaacIcacaWGJbWaaSbaaSqaaiaadQgaaeqaaOGaaiiFaiaadsgadaWgaaWcbaGaamyAaaqabaGccaGGPaGaeyypa0ZaaCbeaeaaciGGHbGaaiOCaiaacEgaciGGTbGaaiyyaiaacIhaaSqaaiaadogadaWgaaadbaGaamOAaaqabaaaleqaaOWaaSaaaeaacaWGqbGaaiikaiaadsgadaWgaaWcbaGaamyAaaqabaGccaGG8bGaam4yamaaBaaaleaacaWGQbaabeaakiaacMcacaWGqbGaaiikaiaadogadaWgaaWcbaGaamOAaaqabaGccaGGPaaabaGaamiuaiaacIcacaWGKbWaaSbaaSqaaiaadMgaaeqaaOGaaiykaaaaaaa@607E@

An accurate Bayesian classifier is dependent on accurate estimates for the probabilities on the right-hand side of equation 1. The denominator *P*(*d*_*i*_) is dropped because it is constant. The prior probability of each subcellular localization *c*_*j*_, denoted *P*(*c*_*j*_), is estimated from D by counting the number of sequences assigned to class *c*_*j*_, divided by the total number of sequences in D:

P(cj)=|Dcj||D|
 MathType@MTEF@5@5@+=feaafiart1ev1aaatCvAUfeBSjuyZL2yd9gzLbvyNv2Caerbhv2BYDwAHbqedmvETj2BSbqee0evGueE0jxyaibaiKI8=vI8tuQ8FMI8Gi=hEeeu0xXdbba9frFj0=OqFfea0dXdd9vqai=hGuQ8kuc9pgc9s8qqaq=dirpe0xb9q8qiLsFr0=vr0=vr0dc8meaabaqaciGacaGaaeqabaqadeqadaaakeaacaWGqbGaaiikaiaadogadaWgaaWcbaGaamOAaaqabaGccaGGPaGaeyypa0ZaaSaaaeaadaabdaqaamrtHrhAL1wy0L2yHvtyaeHbnfgDOvwBHrxAJfwnaGabaiab=nq8enaaBaaaleaacaWGJbWaaSbaaWqaaiaadQgaaeqaaaWcbeaaaOGaay5bSlaawIa7aaqaamaaemaabaGae83aXteacaGLhWUaayjcSdaaaaaa@4E3E@

**Figure 4:**
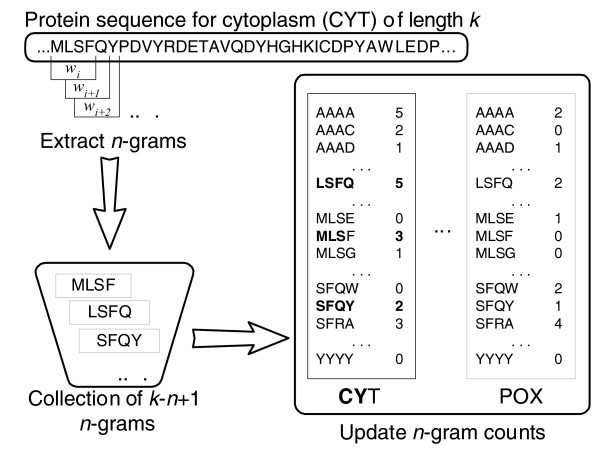
The *n*-gram model for representing proteins in ngLOC. This figure depicts the process of extracting *n*-grams from an example protein sequence for cytoplasm (CYT), and shows how the table of frequencies of *n*-grams maintained by the model is updated accordingly. For this example, *n *= 4. The *n*-grams in bold indicate updated table entries as a result of processing the sequence. POX, perixosome.

*P*(*d*_*i *_| *c*_*j*_), the posterior probability of protein sequence *d*_*i *_given location *c*_*j*_, is the difficult parameter to estimate. As discussed, we use the *n*-gram model to represent proteins. We make the naïve Bayes assumption, in which the occurrence of the *t*^*th *^*n*-gram in S, denoted *w*_*t*_, is identically and independently distributed (IID) with respect to every other *n*-gram in S, where each *w*_*t *_follows a Bernoulli distribution. In reality, this is not a correct assumption, because *n*-grams are clearly not independent of each other. An accurate probabilistic model based on *n*-grams would need to consider a joint distribution over all possible combinations of *n*-grams, which is an impossible task because of the exponential amount of memory required and because sequences vary in length. Fortunately, the IID assumption has worked well in practice. With these assumptions, each sequence *d*_*i *_is viewed as a collection of unordered *n*-grams generated by a random process following a multinomial distribution [36]. Estimating the posterior probabilities for each class simply involves counting *n*-gram occurrences in the dataset for each class separately (Figure 4). If a sequence in the dataset is multi-localized, then it will have multiple class labels. In this case, the count of *n*-grams for each class that sequence is localized to will be updated equally. We let *N*_*it *_be the count of the number of occurrences of *n*-gram *w*_*t *_in sequence *d*_*i*_. Under these distribution assumptions, we can derive an estimate for the probability of a sequence, given a class, as follows:

P(di|cj)=(∑tNit)!∏t(Nit!)∏tP(wt|cj)Nit
MathType@MTEF@5@5@+=feaafiart1ev1aaatCvAUfeBSjuyZL2yd9gzLbvyNv2Caerbhv2BYDwAHbqedmvETj2BSbqee0evGueE0jxyaibaiKI8=vI8tuQ8FMI8Gi=hEeeu0xXdbba9frFj0=OqFfea0dXdd9vqai=hGuQ8kuc9pgc9s8qqaq=dirpe0xb9q8qiLsFr0=vr0=vr0dc8meaabaqaciGacaGaaeqabaqadeqadaaakeaacaWGqbGaaiikaiaadsgadaWgaaWcbaGaamyAaaqabaGccaGG8bGaam4yamaaBaaaleaacaWGQbaabeaakiaacMcacqGH9aqpdaWcaaqaamaabmaabaWaaabeaeaacaWGobWaaSbaaSqaaiaadMgacaWG0baabeaaaeaacaWG0baabeqdcqGHris5aaGccaGLOaGaayzkaaGaaiyiaaqaamaarababaGaaiikaiaad6eadaWgaaWcbaGaamyAaiaadshaaeqaaOGaaiyiaiaacMcaaSqaaiaadshaaeqaniabg+GivdaaaOWaaebeaeaacaWGqbGaaiikaiaadEhadaWgaaWcbaGaamiDaaqabaGccaGG8bGaam4yamaaBaaaleaacaWGQbaabeaakiaacMcadaahaaWcbeqaaiaad6eadaWgaaadbaGaamyAaiaadshaaeqaaaaaaSqaaiaadshaaeqaniabg+Givdaaaa@5882@

The first term is disregarded because of equation 1. The probability of sequence *d*_*i *_given class *c*_*j *_is then estimated as the product of the probabilities of all *n*-grams that occurred in *d*_*i *_for that particular class. To estimate *P*(*w*_*t *_| *c*_*j*_), the posterior probability of *n*-gram *w*_*t *_given class *c*_*j*_, we use the LaPlace correction to prevent zero probabilities from being calculated and count the number of occurrences of *w*_*t *_in all sequences belonging to that class:

P(wt|cj)=1+Count(wt) in class cj|S|+(Total n−grams in class cj)
 MathType@MTEF@5@5@+=feaafiart1ev1aaatCvAUfeBSjuyZL2yd9gzLbvyNv2Caerbhv2BYDwAHbqedmvETj2BSbqee0evGueE0jxyaibaiKI8=vI8tuQ8FMI8Gi=hEeeu0xXdbba9frFj0=OqFfea0dXdd9vqai=hGuQ8kuc9pgc9s8qqaq=dirpe0xb9q8qiLsFr0=vr0=vr0dc8meaabaqaciGacaGaaeqabaqadeqadaaakeaacaWGqbWaaeWaaeaacaWG3bWaaSbaaSqaaiaadshaaeqaaOGaaiiFaiaadogadaWgaaWcbaGaamOAaaqabaaakiaawIcacaGLPaaacqGH9aqpdaWcaaqaaiaaigdacqGHRaWkcaWGdbGaam4BaiaadwhacaWGUbGaamiDaiaacIcacaWG3bWaaSbaaSqaaiaadshaaeqaaOGaaiykaiaabccacaWGPbGaamOBaiaabccacaWGJbGaamiBaiaadggacaWGZbGaam4CaiaabccacaWGJbWaaSbaaSqaaiaadQgaaeqaaaGcbaWaaqWaaeaat0uy0HwzTfgDPnwy1egaryqtHrhAL1wy0L2yHvdaiqaacqWFse=uaiaawEa7caGLiWoacqGHRaWkcaGGOaGaamivaiaad+gacaWG0bGaamyyaGqaciaa+XgacaqGGaGaa4NBaiabgkHiTiaadEgacaWGYbGaamyyaiaad2gacaGFZbGaaeiiaiaa+LgacaGFUbGaaeiiaiaadogacaWGSbGaamyyaiaadohacaWGZbGaaeiiaiaadogadaWgaaWcbaGaamOAaaqabaGccaGGPaaaaaaa@7762@

To prevent loss of precision, we convert the probability in equation 3 to a log-probability, which allows us to take the sum of the log of the probabilities calculated in equation 4.

### Probabilistic confidence score

An advantage in using a probabilistic model, such as a naïve Bayes classifier or a Hidden Markov Model, over nonprobabilistic approaches is that the probability of the model generating a given sequence is inherently reported for every class. Although the prediction is based on the model with the highest probability, the probability can also be used as a comparative measure against other classes. If the predicted class had a low probability, it might suggest that the second or third highest predictions should also be considered. If the top two classes predicted were relatively high compared to the rest of the classes, it might suggest the possibility that the sequence is localized into both locations. We develop a probabilistic CS for sequence *d*_*i *_for each possible class *c*_*j*_, denoted *CS*(*d*_*i *_| *c*_*j*_), and show how this is used to address each of the above issues. We also derive a multi-localized confidence score, denoted *MLCS*(*d*_*i*_), that gives a probabilistic measure of sequence *d*_*i *_being multi-localized.

For a given sequence *d*_*i*_, we define *d*_*null *_to be a sequence of null symbols of a length that is equal to the length of *d*_*i*_. (A null symbol can be any symbol *s *such that s ∉ Σ. Each *n*-gram in *d*_*null *_is guaranteed never to occur in the model. To calculate the probability that each class generated *d*_*null*_, we can use this fact to simplify the calculations of equations 3 and 4, giving us:

P(dnull|cj)=P(1|S|+(Total n−grams in class cj)(k−n+1)
 MathType@MTEF@5@5@+=feaafiart1ev1aaatCvAUfeBSjuyZL2yd9gzLbvyNv2Caerbhv2BYDwAHbqedmvETj2BSbqee0evGueE0jxyaibaiKI8=vI8tuQ8FMI8Gi=hEeeu0xXdbba9frFj0=OqFfea0dXdd9vqai=hGuQ8kuc9pgc9s8qqaq=dirpe0xb9q8qiLsFr0=vr0=vr0dc8meaabaqaciGacaGaaeqabaqadeqadaaakeaacaWGqbWaaeWaaeaacaWGKbWaaSbaaSqaaiaad6gacaWG1bGaamiBaiaadYgaaeqaaOGaaiiFaiaadogadaWgaaWcbaGaamOAaaqabaaakiaawIcacaGLPaaacqGH9aqpcaWGqbWaaeWaaeaadaWcaaqaaiaaigdaaeaadaabdaqaamrtHrhAL1wy0L2yHvtyaeHbnfgDOvwBHrxAJfwnaGabaiab=jr8tbGaay5bSlaawIa7aiabgUcaRiaacIcacaWGubGaam4BaiaadshacaWGHbacbiGaa4hBaiaabccacaGFUbGaeyOeI0Iaam4zaiaadkhacaWGHbGaamyBaiaa+nhacaqGGaGaa4xAaiaa+5gacaqGGaGaam4yaiaadYgacaWGHbGaam4CaiaadohacaqGGaGaam4yamaaBaaaleaacaWGQbaabeaaaaaakiaawIcacaGLPaaadaahaaWcbeqaamaabmaabaGaam4AaiabgkHiTiaad6gacqGHRaWkcaaIXaaacaGLOaGaayzkaaaaaaaa@6E6B@

We then set *minNullProb *to be the minimum joint probability of *d*_*null *_and class *c*_*j *_across all classes:

minNullProb=min⁡cj∈C(P(cj)P(dnull|cj))
 MathType@MTEF@5@5@+=feaafiart1ev1aaatCvAUfeBSjuyZL2yd9gzLbvyNv2Caerbhv2BYDwAHbqedmvETj2BSbqee0evGueE0jxyaibaiKI8=vI8tuQ8FMI8Gi=hEeeu0xXdbba9frFj0=OqFfea0dXdd9vqai=hGuQ8kuc9pgc9s8qqaq=dirpe0xb9q8qiLsFr0=vr0=vr0dc8meaabaqaciGacaGaaeqabaqadeqadaaakeaaieGacaWFTbGaa8xAaiaa=5gacaWGobGaamyDaiaadYgacaWGSbGaa8huaiaa=jhacaWGVbGaamOyaiabg2da9maaxababaGaciyBaiaacMgacaGGUbaaleaacaWGJbWaaSbaaWqaaiaadQgaaeqaaSGaeyicI48enfgDOvwBHrxAJfwnHbqeg0uy0HwzTfgDPnwy1aaceaGae4NaXpeabeaakmaabmaabaGaamiuamaabmaabaGaam4yamaaBaaaleaacaWGQbaabeaaaOGaayjkaiaawMcaaiaadcfadaqadaqaaiaadsgadaWgaaWcbaGaamOBaiaadwhacaWGSbGaamiBaaqabaGccaGG8bGaam4yamaaBaaaleaacaWGQbaabeaaaOGaayjkaiaawMcaaaGaayjkaiaawMcaaaaa@60BF@

A log-odds ratio that sequence *d*_*i *_is targeted for location *c*_*j *_against *minNullProb *is calculated and then normalized by dividing by the sum over all log-odds scores, to create a separate score for each subcellular location *c*_*j *_for a given sequence *d*_*i *_as follows:

CS(cj|di)=log⁡(P(cj)P(di|cj))−log⁡(minNullProb)∑k(log⁡(P(ck)P(di|ck))−log⁡(minNullProb))∗100
 MathType@MTEF@5@5@+=feaafiart1ev1aaatCvAUfeBSjuyZL2yd9gzLbvyNv2Caerbhv2BYDwAHbqedmvETj2BSbqee0evGueE0jxyaibaiKI8=vI8tuQ8FMI8Gi=hEeeu0xXdbba9frFj0=OqFfea0dXdd9vqai=hGuQ8kuc9pgc9s8qqaq=dirpe0xb9q8qiLsFr0=vr0=vr0dc8meaabaqaciGacaGaaeqabaqadeqadaaakeaacaWGdbGaam4uamaabmaabaGaam4yamaaBaaaleaacaWGQbaabeaakiaacYhacaWGKbWaaSbaaSqaaiaadMgaaeqaaaGccaGLOaGaayzkaaGaeyypa0ZaaSaaaeaaciGGSbGaai4BaiaacEgadaqadaqaaiaadcfadaqadaqaaiaadogadaWgaaWcbaGaamOAaaqabaaakiaawIcacaGLPaaacaWGqbWaaeWaaeaacaWGKbWaaSbaaSqaaiaadMgaaeqaaOGaaiiFaiaadogadaWgaaWcbaGaamOAaaqabaaakiaawIcacaGLPaaaaiaawIcacaGLPaaacqGHsislciGGSbGaai4BaiaacEgadaqadaqaaGqaciaa=1gacaWFPbGaa8NBaiaad6eacaWG1bGaamiBaiaadYgacaWFqbGaa8NCaiaad+gacaWGIbaacaGLOaGaayzkaaaabaWaaabeaeaadaqadaqaaiGacYgacaGGVbGaai4zamaabmaabaGaamiuamaabmaabaGaam4yamaaBaaaleaacaWGRbaabeaaaOGaayjkaiaawMcaaiaadcfadaqadaqaaiaadsgadaWgaaWcbaGaamyAaaqabaGccaGG8bGaam4yamaaBaaaleaacaWGRbaabeaaaOGaayjkaiaawMcaaaGaayjkaiaawMcaaiabgkHiTiGacYgacaGGVbGaai4zamaabmaabaGaa8xBaiaa=LgacaWFUbGaamOtaiaadwhacaWGSbGaamiBaiaa=bfacaWFYbGaam4BaiaadkgaaiaawIcacaGLPaaaaiaawIcacaGLPaaaaSqaaiaadUgaaeqaniabggHiLdaaaOGaey4fIOIaaGymaiaaicdacaaIWaaaaa@8345@

The range for each score will always be between 0 and 100, with the sum of the scores over all classes totaling 100. This CS can also be interpreted as an estimate of the conditional probability of class *c*_*j*_, given sequence *d*_*i *_and the *n*-gram model used.

The multi-localized confidence score for sequence *d*_*i *_(equation 8) is derived from the CSs of the two most probable classes for that sequence, denoted as *CS*_1 _and *CS*_2_, respectively. This score is designed to give a relative measure of the likelihood that sequence *d*_*i *_is multi-localized into two organelles.

MLCS(di)=(CS1+CS2)−(CS12−CS22)100.0
 MathType@MTEF@5@5@+=feaafiart1ev1aaatCvAUfeBSjuyZL2yd9gzLbvyNv2Caerbhv2BYDwAHbqedmvETj2BSbqee0evGueE0jxyaibaiKI8=vI8tuQ8FMI8Gi=hEeeu0xXdbba9frFj0=OqFfea0dXdd9vqai=hGuQ8kuc9pgc9s8qqaq=dirpe0xb9q8qiLsFr0=vr0=vr0dc8meaabaqaciGacaGaaeqabaqadeqadaaakeaacaWGnbGaamitaiaadoeacaWGtbWaaeWaaeaacaWGKbWaaSbaaSqaaiaadMgaaeqaaaGccaGLOaGaayzkaaGaeyypa0ZaaeWaaeaacaWGdbGaam4uamaaBaaaleaacaaIXaaabeaakiabgUcaRiaadoeacaWGtbWaaSbaaSqaaiaaikdaaeqaaaGccaGLOaGaayzkaaGaeyOeI0YaaSaaaeaadaqadaqaaiaadoeacaWGtbWaa0baaSqaaiaaigdaaeaacaaIYaaaaOGaeyOeI0Iaam4qaiaadofadaqhaaWcbaGaaGOmaaqaaiaaikdaaaaakiaawIcacaGLPaaaaeaacaaIXaGaaGimaiaaicdacaGGUaGaaGimaaaaaaa@5045@

### Application of ngLOC to proteome-wide predictions

Most protein classification models, including ngLOC, are built using datasets from sequences over many species across the eukaryotic superkingdom. In fact, the ngLOC dataset exhibited in Table 9 contains proteins from 1,923 different species. This introduces another variable that can be observed. Although some methods indirectly observe the relationship between the species and the dependent variable by incorporating phylogenic information in their model, it is usually not directly observed. However, it is known that in the case of subcellular localization the distribution between classes varies among species. For example, one study of mitochondrial proteins estimates that 9.9% of the yeast proteome is localized in the mitochondria, as compared with an estimated 4.8% of the human proteome [16]. Another study estimated that as much as 13% of the yeast proteome is localized in the mitochondria [25]. It is clear that the predictions for the proteome of a specific species can be improved if the prior probability P(*c*_*j*_) is known for the species being predicted. Unfortunately, this information is not available before classification.

We extend equation 1 by introducing another random variable *X *that will represent the species being predicted. We will refer to the model that incorporates the proteome in this manner as ngLOC-X:

P(cj|di,X)=P(di|cj,X)P(cj|X)P(di|X)
 MathType@MTEF@5@5@+=feaafiart1ev1aaatCvAUfeBSjuyZL2yd9gzLbvyNv2Caerbhv2BYDwAHbqedmvETj2BSbqee0evGueE0jxyaibaiKI8=vI8tuQ8FMI8Gi=hEeeu0xXdbba9frFj0=OqFfea0dXdd9vqai=hGuQ8kuc9pgc9s8qqaq=dirpe0xb9q8qiLsFr0=vr0=vr0dc8meaabaqaciGacaGaaeqabaqadeqadaaakeaacaWGqbWaaeWaaeaacaWGJbWaaSbaaSqaaiaadQgaaeqaaOGaaiiFaiaadsgadaWgaaWcbaGaamyAaaqabaGccaGGSaGaamiwaaGaayjkaiaawMcaaiabg2da9maalaaabaGaamiuamaabmaabaGaamizamaaBaaaleaacaWGPbaabeaakiaacYhacaWGJbWaaSbaaSqaaiaadQgaaeqaaOGaaiilaiaadIfaaiaawIcacaGLPaaacaWGqbWaaeWaaeaacaWGJbWaaSbaaSqaaiaadQgaaeqaaOGaaiiFaiaadIfaaiaawIcacaGLPaaaaeaacaWGqbWaaeWaaeaacaWGKbWaaSbaaSqaaiaadMgaaeqaaOGaaiiFaiaadIfaaiaawIcacaGLPaaaaaaaaa@52DD@

We only need to consider the two terms in the denominator for reasons stated previously. We solve for *P*(*d*_*i *_| *c*_*j*_, *X*), the probability of a protein sequence, given subcellular localization *c*_*j *_and species *X*, by assuming a mixture model of two independent conditional probability distributions over the space of protein sequences. One distribution is over proteins with known subcellular localization but unknown species, and the other distribution is over proteins of a known species but unknown subcellular localization. This forms a mixture model of two distributions, formally stated as follows:

*P*(*d*_*i *_| *c*_*j*_, *X*) = α_*j *_*P*(*d*_*i *_| *c*_*j*_) + (1 - α_*j*_) *P*(*d*_*i *_| *X*)

The component P(*d*_*i *_| *c*_*j*_) is estimated as given in equation 3. The component *P*(*d*_*i *_| *X*) is estimated in the same way; however, we let *N*_*it *_be the count of the number of occurrences of *n*-gram *w*_*t *_in the proteome for species X, and *P*(*w*_*t *_| *c*_*j*_) is replaced by *P*(*w*_*t *_| *X*), which is the probability of *n*-gram *w*_*t*_, given species *X*. To estimate α, the mixture proportion for P(*d*_*i *_| *c*_*j*_), we use the following:

αj=(Total n−grams in class cj)(Total n−grams inclass cj)+(Total n−grams in species X)
 MathType@MTEF@5@5@+=feaafiart1ev1aaatCvAUfeBSjuyZL2yd9gzLbvyNv2Caerbhv2BYDwAHbqedmvETj2BSbqee0evGueE0jxyaibaiKI8=vI8tuQ8FMI8Gi=hEeeu0xXdbba9frFj0=OqFfea0dXdd9vqai=hGuQ8kuc9pgc9s8qqaq=dirpe0xb9q8qiLsFr0=vr0=vr0dc8meaabaqaciGacaGaaeqabaqadeqadaaakeaacqaHXoqydaWgaaWcbaGaamOAaaqabaGccqGH9aqpdaWcaaqaamaabmaabaGaamivaiaad+gacaWG0bGaamyyaGqaciaa=XgacaqGGaGaa8NBaiabgkHiTiaadEgacaWGYbGaamyyaiaad2gacaWFZbGaaeiiaiaa=LgacaWFUbGaaeiiaiaadogacaWGSbGaamyyaiaadohacaWGZbGaaeiiaiaadogadaWgaaWcbaGaamOAaaqabaaakiaawIcacaGLPaaaaeaadaqadaqaaiaadsfacaWGVbGaamiDaiaadggacaWFSbGaaeiiaiaa=5gacqGHsislcaWGNbGaamOCaiaadggacaWGTbGaa83CaiaabccacaWFPbGaa8NBaiaa=bcacaWGJbGaamiBaiaadggacaWGZbGaam4CaiaabccacaWGJbWaaSbaaSqaaiaadQgaaeqaaaGccaGLOaGaayzkaaGaey4kaSYaaeWaaeaacaWGubGaam4BaiaadshacaWGHbGaa8hBaiaabccacaWFUbGaeyOeI0Iaam4zaiaadkhacaWGHbGaamyBaiaa=nhacaqGGaGaa8xAaiaa=5gacaqGGaGaam4CaiaadchacaWGLbGaam4yaiaadMgacaWGLbGaam4CaiaabccacaWGybaacaGLOaGaayzkaaaaaaaa@800C@

The mixture model allows us to consider the distribution of *n*-grams over an entire proteome by adjusting the probabilities of each *n*-gram in the training data to represent more accurately the distributions of *n*-grams in the proteome being classified. The result is that the distribution of *P*(*w*_*t *_| *c*_*j*_) over all *n*-grams will be more similar to that of the proteome, while retaining the relative probabilities of each class within individual *n*-grams learned from the labeled data.

Solving for *P*(*c*_*j *_| *X*) is difficult because we do not know the distribution of subcellular localizations in a given species, and neither can it be observed before classification. However, we incorporate the proteome for species *X *using a LaPlacean-type of estimate, adding the proteome data to each class. The result is shown in equation 12, where D_*x *_represents the dataset consisting of sequences from the proteome of species *X*:

P(cj|X)=|Dcj|+|DX||D|+(|C||DX|)
 MathType@MTEF@5@5@+=feaafiart1ev1aaatCvAUfeBSjuyZL2yd9gzLbvyNv2Caerbhv2BYDwAHbqedmvETj2BSbqee0evGueE0jxyaibaiKI8=vI8tuQ8FMI8Gi=hEeeu0xXdbba9frFj0=OqFfea0dXdd9vqai=hGuQ8kuc9pgc9s8qqaq=dirpe0xb9q8qiLsFr0=vr0=vr0dc8meaabaqaciGacaGaaeqabaqadeqadaaakeaacaWGqbWaaeWaaeaacaWGJbWaaSbaaSqaaiaadQgaaeqaaOGaaiiFaiaadIfaaiaawIcacaGLPaaacqGH9aqpdaWcaaqaamaaemaabaWenfgDOvwBHrxAJfwnHbqeg0uy0HwzTfgDPnwy1aaceaGae83aXt0aaSbaaSqaaiaadogadaWgaaadbaGaamOAaaqabaaaleqaaaGccaGLhWUaayjcSdGaey4kaSYaaqWaaeaacqWFdeprdaWgaaWcbaGaamiwaaqabaaakiaawEa7caGLiWoaaeaadaabdaqaaiab=nq8ebGaay5bSlaawIa7aiabgUcaRmaabmaabaWaaqWaaeaacqWFce=qaiaawEa7caGLiWoadaabdaqaaiab=nq8enaaBaaaleaacaWGybaabeaaaOGaay5bSlaawIa7aaGaayjkaiaawMcaaaaaaaa@6459@

This method can cause the probability estimates for *P*(*c*_*j *_| *X*) to become more uniform in proportion to the size of the dataset of the proteome (D_*x*_) being considered. We accept this tendency, because it implicitly factors in a measure of uncertainty proportional to the size of the proteome being considered, meaning the larger the proteome, the more uncertainty there is in regard to the exact prior probabilities of each subcellular localization. In this case, the priors should not be based solely on the exact percentages of the ngLOC training data.

## Additional data files

The following additional data files are available with the online version of the paper. Additional data file 1 contains all of the formulas used for performance measurements and Supplementary Tables 1-21. Additional data file 2 contains the actual ngLOC dataset.

## Supplementary Material

Additional data file 1 Provided are all of the formulas used for performance measurements and Supplementary Tables 1-21. Click here for file

Additional data file 2 Provided is the actual ngLOC dataset. It is a FASTA formatted file. The header format for each sequence is > SP_name loc [/loc2], where SP_name is the Swiss-Prot name of the protein sequence, from release 50.0, loc is a single letter representing subcellular localization for this sequence, and/loc2 is an optional field that exists only if the sequence is multi-localized. The letter codes for subcellular localization are as follows: C (CYT), cytoplasm; K (CSK), cytoskeleton[E (END), endoplasmic reticulum; S (EXC), extracellular/secreted; G (GOL), Golgi; L (LYS), lysosome; M (MIT), mitochondria; N (NUC), nucleus; P (PLA), plasma membrane; X (POX), perixosome. Click here for file
