# Supplementary material for: ngLOC: an n-gram-based Bayesian method for estimating the subcellular proteomes of eukaryotes
Source: Genome Biol. 2007 May 1;8(5):R68. doi: 10.1186/gb-2007-8-5-r68 (PMC1929137; doi:10.1186/gb-2007-8-5-r68)
Supplement: Additional data file 1 — Provided are all of the formulas used for performance measurements and Supplementary Tables 1-21. [file gb-2007-8-5-r68-S1.doc]

# Performance Measurements

For each subcellular localization class *c_j_*, we maintain counts of the following:

- True positives (*TP_j_*) – the number of sequences correctly predicted to localize into *c_j_*.
- True negatives (*TN_j_*) – the number of sequences correctly predicted to not localize into *c_j_*.
- False positives (*FP_j_*) – the number of sequences predicted to localize into *c_j_*, but actually localize elsewhere.
- False negatives (*FN_j_*) – the number of sequences that actually localize into *c_j_*, but were predicted to localize elsewhere.

Using these four quantitative measures, we report a number of standard measurements in judging classifier performance:

- *Overall Accuracy* – a measure of the overall classifier performance. It is defined as the fraction of the data tested that is classified correctly. Though it is a poor measure to consider on highly unbalanced datasets, we still report it as a general overall comparative measure:

- *Sensitivity* (a.k.a. *Recall*, *TP-rate*) – the fraction of data in class *c_j_* that was correctly predicted. This gives a measure of individual class accuracy:

- *Precision* – the fraction of data predicted to be in class *c_j_* that was actually correct:

$Prec_{j}=\frac{TP_{j}}{TP_{j}+FP_{j}}$

- *Specificity* – the fraction of data not in class *c_j_* that was correctly predicted:

- *False Positive Rate* – the fraction of data not in class *c_j_* that was incorrectly predicted to be in class *c_j_*:

- *Matthews Correlation Coefficient* – a measure of overall predictive performance for class *c_j_*. It has a range of (-1,+1), where 1 implies a perfect prediction, 0 implies random, and a value of less than 0 implies that the prediction is worse than random guessing:

$$MCC_{j}=\frac{TP_{j}TN_{j}-FP_{j}FN_{j}}{\sqrt{\left(TP_{j}+FP_{j}\right)\left(TP_{j}+FN_{j}\right)\left(TN_{j}+FP_{j}\right)\left(TN_{j}+FN_{j}\right)}}$$

Table 1 – Examples of outdated annotations in the PLOC dataset discovered by ngLOC

| **SP ID** | **PLOC**  **Anotation** | **Correct** | **ngLOC**  **Predict** | **CYT** | **CSK** | **END** | **EXC** | **GOL** | **LYS** | **MIT** | **NUC** | **PLA** | **POX** |
| --- | --- | --- | --- | --- | --- | --- | --- | --- | --- | --- | --- | --- | --- |
| P40541 | CYT | NUC | NUC | 1.2 | 0.2 | 0.5 | 0.7 | 0.3 | 0.0 | 0.4 | **96.1** | 0.5 | 0.0 |
| Q03562 | PLA | CYT | CYT | **95.5** | 0.3 | 0.5 | 1.0 | 0.3 | 0.0 | 0.7 | 0.9 | 0.7 | 0.1 |
| P32522 | PLA | MIT | MIT | 0.8 | 0.1 | 0.3 | 0.9 | 0.1 | 0.2 | **95.5** | 1.4 | 0.8 | 0.0 |
| P33287 | CSK | CYT | CYT | **94.9** | 0.2 | 0.7 | 0.9 | 0.2 | 0.0 | 0.7 | 0.9 | 1.3 | 0.1 |
| P53730 | PLA | END | END | 1.1 | 0.2 | **94.5** | 0.8 | 0.2 | 0.0 | 0.8 | 1.0 | 1.4 | 0.1 |

This table shows some examples of sequences in the PLOC dataset that had outdated annotations, that were discovered by ngLOC.

Table 2 – Examples of multi-localized sequences in the PLOC dataset discovered by ngLOC

| **SP ID** | **PLOC**  **Anotation** | **Correct** | **ngLOC**  **Predict** | **MLCS** | **CYT** | **CSK** | **END** | **EXC** | **GOL** | **LYS** | **MIT** | **NUC** | **PLA** | **POX** |
| --- | --- | --- | --- | --- | --- | --- | --- | --- | --- | --- | --- | --- | --- | --- |
| P97840 | CYT | CYT/EXC | EXC | **90.0** | **45.0** | 0.5 | 1.0 | **45.4** | 0.5 | 0.0 | 1.4 | 3.5 | 2.7 | 0.1 |
| P70583 | CYT | CYT/NUC | NUC | **74.2** | **36.3** | 0.2 | 0.5 | 1.1 | 0.2 | 0.0 | 15.5 | **45.1** | 1.0 | 0.1 |
| P01095 | CYT | CYT/NUC | NUC | **87.8** | **43.9** | 0.8 | 1.6 | 3.2 | 0.9 | 0.0 | 2.3 | **44.1** | 3.0 | 0.2 |

Here, we present some examples of multi-localized sequences in the PLOC dataset that were discovered by ngLOC.

Table 3 – Example misclassifications of ngLOC method when training and testing on PLOC dataset

| **SP ID** | **PLOC**  **Annotation** | **ngLOC**  **Predict** | **CHL** | **CYT** | **CSK** | **END** | **EXC** | **GOL** | **LYS** | **MIT** | **NUC** | **PLA** | **POX** | **VAC** |
| --- | --- | --- | --- | --- | --- | --- | --- | --- | --- | --- | --- | --- | --- | --- |
| P81649 | LYS | EXC | 2.7 | 1.4 | 3.4 | 3.3 | **37.7** | 3.4 | **32.5** | 4.1 | 1.5 | 3.4 | 3.3 | 3.4 |
| P21954 | MIT | CYT | 15.6 | **37.7** | 1.8 | 1.9 | 1.7 | 1.4 | 1.3 | **31.0** | 2.5 | 2.2 | 1.5 | 1.4 |
| P51242 | CHL | MIT | **34.3** | 4.1 | 1.9 | 1.9 | 2.0 | 1.9 | 2.3 | **37.3** | 5.5 | 4.2 | 2.0 | 2.7 |
| P05123 | CYT | MIT | 5.5 | **16.9** | 4.4 | 3.8 | 3.9 | 3.9 | 5.8 | **39.4** | 2.8 | 6.0 | 3.7 | 3.9 |
| P30412 | CYT | END | 14.7 | **22.5** | 2.1 | **27.0** | 9.3 | 1.6 | 2.5 | 8.6 | 3.3 | 4.7 | 1.6 | 2.1 |

This table shows examples of sequences the ngLOC method misclassified, using a 6-gram model trained on the PLOC dataset, using all 12 possible localization classes, as annotated in the PLOC data. For each sequence, the top two predictions are in bold. This shows how the confidence score can be used to consider possible alternatives when the confidence score is low, as the correct prediction was the second highest.

Table 4 – ngLOC subcellular estimates for eight proteomes (actual numbers)

|  | Yeast | Worm | Fruitfly | Mosquito | Zebrafish | Chicken | Mouse | Human |
| --- | --- | --- | --- | --- | --- | --- | --- | --- |
|  | *S.cerevisiae* | *C.elegans* | *D.melano.* | *A.gambiae* | *D.rerio* | *G.gallus* | *M.musculus* | *H.sapiens* |
| Proteome: | 5799 | 22400 | 13649 | 15145 | 13803 | 5394 | 33043 | 38149 |
| GO annotated: | 5486 | 12357 | 9997 | 8847 | 10106 | 4363 | 23744 | 24638 |
| % ngLOC Coverage: | 97.48 | 94.92 | 96.73 | 97.94 | 98.64 | 99.82 | 94.79 | 94.52 |
| Proteome Est: | 5653 | 21262 | 13203 | 14833 | 13616 | 5384 | 31320 | 36059 |
| CYT | 860 | 3147 | 1682 | 2140 | 2043 | 736 | 4210 | 5097 |
| END | 153 | 738 | 377 | 483 | 455 | 136 | 937 | 1095 |
| GOL | 84 | 279 | 184 | 159 | 228 | 79 | 477 | 564 |
| CSK | 60 | 252 | 139 | 164 | 179 | 67 | 469 | 533 |
| LYS | 6 | 123 | 72 | 79 | 89 | 23 | 184 | 243 |
| MIT | 540 | 1242 | 642 | 818 | 643 | 224 | 1329 | 1730 |
| NUC | 1895 | 6325 | 4936 | 4376 | 4128 | 1521 | 8566 | 10235 |
| PLA | 915 | 5190 | 2649 | 3169 | 2949 | 1226 | 8511 | 8682 |
| EXC | 502 | 2679 | 1619 | 2118 | 1349 | 681 | 3609 | 4224 |
| POX | 31 | 140 | 56 | 72 | 70 | 14 | 138 | 167 |
| Single-Localized: | 5047 | 20114 | 12356 | 13577 | 12133 | 4707 | 28428 | 32569 |
| Multi-Localized: | 606 | 1147 | 847 | 1257 | 1483 | 677 | 2892 | 3490 |
| CYT-NUC: | 367 | 502 | 365 | 510 | 735 | 338 | 1411 | 1708 |

Table 5 – Analysis of discriminatory n-grams in domain regions

| **Location** | **Expected** | **Observed** |
| --- | --- | --- |
| CYT | 77.5 | 96.9 |
| CSK | 65.9 | 85.3 |
| END | 83.4 | 96.0 |
| EXC | 80.8 | 89.5 |
| GOL | 73.1 | 97.5 |
| LYS | 86.9 | 96.7 |
| MIT | 86.1 | 97.8 |
| NUC | 53.7 | 80.2 |
| PLA | 80.0 | 92.1 |
| POX | 85.0 | 97.4 |
| **TOTAL** | **75.5** | **91.4** |

This table shows the comparison between the expected percentage of *n*-grams that will be found in a domain region over all sequences in each class, against the observed percentage of discriminatory *n*-grams that were found in those same domain regions. Though 75.5% of all n-grams were found in a domain region, we observed 91.4% of all discriminatory *n*-grams to be located in these same domain regions. These results were calculated over 15,109 protein sequences in the ngLOC dataset that had at least one Interpro domain definition mapping available.

**HUMAN (*H.sapiens*)**

Table 6 – Subcellular proteome percentage estimates for human

|  | **CYT** | **CSK** | **END** | **EXC** | **GOL** | **LYS** | **MIT** | **NUC** | **PLA** | **POX** |
| --- | --- | --- | --- | --- | --- | --- | --- | --- | --- | --- |
| **CYT** | 14.14 |  |  |  |  |  |  |  |  |  |
| **CSK** | 0.64 | 1.48 |  |  |  |  |  |  |  |  |
| **END** | 0.10 | 0.01 | 3.04 |  |  |  |  |  |  |  |
| **EXC** | 0.22 | 0.01 | 0.04 | 11.71 |  |  |  |  |  |  |
| **GOL** | 0.29 | 0.03 | 0.31 | 0.17 | 1.56 |  |  |  |  |  |
| **LYS** | 0.02 |  |  | 0.03 | < 0.01 | 0.67 |  |  |  |  |
| **MIT** | 0.31 |  | 0.07 | 0.02 | < 0.01 |  | 4.80 |  |  |  |
| **NUC** | 4.74 | 0.07 | 0.09 | 0.12 | 0.01 |  | 0.09 | 28.38 |  |  |
| **PLA** | 0.77 | 0.02 | 0.14 | 0.94 | 0.09 | 0.00 | 0.03 | 0.19 | 24.08 |  |
| **POX** | 0.05 |  |  | < 0.01 |  |  | 0.03 |  |  | 0.46 |

Table 7 – Subcellular proteome numeric estimates for human

|  | **CYT** | **CSK** | **END** | **EXC** | **GOL** | **LYS** | **MIT** | **NUC** | **PLA** | **POX** |
| --- | --- | --- | --- | --- | --- | --- | --- | --- | --- | --- |
| **CYT** | 5097 |  |  |  |  |  |  |  |  |  |
| **CSK** | 231 | 533 |  |  |  |  |  |  |  |  |
| **END** | 36 | 3 | 1095 |  |  |  |  |  |  |  |
| **EXC** | 79 | 5 | 15 | 4224 |  |  |  |  |  |  |
| **GOL** | 105 | 9 | 113 | 60 | 564 |  |  |  |  |  |
| **LYS** | 8 |  |  | 12 | 2 | 243 |  |  |  |  |
| **MIT** | 113 |  | 25 | 8 | 2 |  | 1730 |  |  |  |
| **NUC** | 1708 | 25 | 31 | 42 | 5 |  | 31 | 10235 |  |  |
| **PLA** | 279 | 8 | 50 | 341 | 34 | 2 | 12 | 70 | 8682 |  |
| **POX** | 17 |  |  | 2 |  |  | 11 |  |  | 167 |

**MOUSE (*M.Musculus*)**

Table 8 – Subcellular proteome percentage estimates for mouse

|  | **CYT** | **CSK** | **END** | **EXC** | **GOL** | **LYS** | **MIT** | **NUC** | **PLA** | **POX** |
| --- | --- | --- | --- | --- | --- | --- | --- | --- | --- | --- |
| **CYT** | 13.44 |  |  |  |  |  |  |  |  |  |
| **CSK** | 0.64 | 1.50 |  |  |  |  |  |  |  |  |
| **END** | 0.08 |  | 2.99 |  |  |  |  |  |  |  |
| **EXC** | 0.19 | < 0.01 | 0.04 | 11.52 |  |  |  |  |  |  |
| **GOL** | 0.27 | 0.04 | 0.32 | 0.12 | 1.52 |  |  |  |  |  |
| **LYS** | 0.03 |  | 0.01 | 0.04 | 0.01 | 0.59 |  |  |  |  |
| **MIT** | 0.31 |  | 0.07 | 0.05 | 0.01 |  | 4.24 |  |  |  |
| **NUC** | 4.51 | 0.08 | 0.09 | 0.16 | 0.02 |  | 0.11 | 27.35 |  |  |
| **PLA** | 0.65 | 0.03 | 0.08 | 0.76 | 0.10 | 0.01 | 0.05 | 0.26 | 27.18 |  |
| **POX** | 0.08 |  |  |  |  |  | 0.01 | < 0.01 | 0.01 | 0.44 |

Table 9 – Subcellular proteome numeric estimates for mouse

|  | **CYT** | **CSK** | **END** | **EXC** | **GOL** | **LYS** | **MIT** | **NUC** | **PLA** | **POX** |
| --- | --- | --- | --- | --- | --- | --- | --- | --- | --- | --- |
| **CYT** | 4210 |  |  |  |  |  |  |  |  |  |
| **CSK** | 200 | 469 |  |  |  |  |  |  |  |  |
| **END** | 26 |  | 937 |  |  |  |  |  |  |  |
| **EXC** | 58 | 1 | 13 | 3609 |  |  |  |  |  |  |
| **GOL** | 85 | 11 | 99 | 38 | 477 |  |  |  |  |  |
| **LYS** | 8 |  | 4 | 11 | 3 | 184 |  |  |  |  |
| **MIT** | 96 |  | 21 | 15 | 3 |  | 1329 |  |  |  |
| **NUC** | 1411 | 25 | 29 | 51 | 6 |  | 35 | 8566 |  |  |
| **PLA** | 203 | 10 | 25 | 238 | 31 | 4 | 17 | 82 | 8511 |  |
| **POX** | 25 |  |  |  |  |  | 3 | 1 | 3 | 138 |

**CHICKEN (*G.gallus*)**

Table 10 – Subcellular proteome percentage estimates for chicken

|  | **CYT** | **CSK** | **END** | **EXC** | **GOL** | **LYS** | **MIT** | **NUC** | **PLA** | **POX** |
| --- | --- | --- | --- | --- | --- | --- | --- | --- | --- | --- |
| **CYT** | 13.66 |  |  |  |  |  |  |  |  |  |
| **CSK** | 1.01 | 1.24 |  |  |  |  |  |  |  |  |
| **END** | 0.21 |  | 2.53 |  |  |  |  |  |  |  |
| **EXC** | 0.23 |  | 0.07 | 12.65 |  |  |  |  |  |  |
| **GOL** | 0.32 | 0.02 | 0.48 | 0.25 | 1.47 |  |  |  |  |  |
| **LYS** | 0.02 |  |  | 0.05 |  | 0.44 |  |  |  |  |
| **MIT** | 0.28 |  | 0.07 | 0.02 | 0.05 |  | 4.16 |  |  |  |
| **NUC** | 6.27 | 0.07 | 0.02 | 0.11 |  |  | 0.14 | 28.24 |  |  |
| **PLA** | 0.90 | 0.02 | 0.11 | 1.17 | 0.14 |  | 0.02 | 0.39 | 22.78 |  |
| **POX** | 0.09 |  |  |  |  |  | 0.02 |  | 0.02 | 0.25 |

Table 11 – Subcellular proteome numeric estimates for chicken

|  | **CYT** | **CSK** | **END** | **EXC** | **GOL** | **LYS** | **MIT** | **NUC** | **PLA** | **POX** |
| --- | --- | --- | --- | --- | --- | --- | --- | --- | --- | --- |
| **CYT** | 736 |  |  |  |  |  |  |  |  |  |
| **CSK** | 54 | 67 |  |  |  |  |  |  |  |  |
| **END** | 11 |  | 136 |  |  |  |  |  |  |  |
| **EXC** | 12 |  | 4 | 681 |  |  |  |  |  |  |
| **GOL** | 17 | 1 | 26 | 14 | 79 |  |  |  |  |  |
| **LYS** | 1 |  |  | 2 |  | 23 |  |  |  |  |
| **MIT** | 15 |  | 4 | 1 | 2 |  | 224 |  |  |  |
| **NUC** | 338 | 4 | 1 | 6 | 0 |  | 7 | 1521 |  |  |
| **PLA** | 48 | 1 | 6 | 63 | 7 |  | 1 | 21 | 1226 |  |
| **POX** | 5 |  |  |  |  |  | 1 |  | 1 | 14 |

**ZEBRAFISH (*D.rerio*)**

Table 12 – Subcellular proteome percentage estimates for zebrafish

|  | **CYT** | **CSK** | **END** | **EXC** | **GOL** | **LYS** | **MIT** | **NUC** | **PLA** | **POX** |
| --- | --- | --- | --- | --- | --- | --- | --- | --- | --- | --- |
| **CYT** | 15.01 |  |  |  |  |  |  |  |  |  |
| **CSK** | 0.81 | 1.31 |  |  |  |  |  |  |  |  |
| **END** | 0.15 |  | 3.34 |  |  |  |  |  |  |  |
| **EXC** | 0.33 | 0.01 | 0.03 | 9.91 |  |  |  |  |  |  |
| **GOL** | 0.17 | 0.02 | 0.40 | 0.17 | 1.68 |  |  |  |  |  |
| **LYS** | 0.02 |  |  | 0.07 | 0.01 | 0.65 |  |  |  |  |
| **MIT** | 0.39 |  | 0.09 | 0.07 | 0.03 |  | 4.72 |  |  |  |
| **NUC** | 5.40 | 0.03 | 0.04 | 0.25 | 0.01 |  | 0.14 | 30.31 |  |  |
| **PLA** | 0.87 | 0.02 | 0.04 | 0.77 | 0.11 |  | 0.09 | 0.27 | 21.66 |  |
| **POX** | 0.06 |  |  |  |  |  | 0.01 |  |  | 0.51 |

Table 13 – Subcellular proteome numeric estimates for zebrafish

|  | **CYT** | **CSK** | **END** | **EXC** | **GOL** | **LYS** | **MIT** | **NUC** | **PLA** | **POX** |
| --- | --- | --- | --- | --- | --- | --- | --- | --- | --- | --- |
| **CYT** | 2043 |  |  |  |  |  |  |  |  |  |
| **CSK** | 111 | 179 |  |  |  |  |  |  |  |  |
| **END** | 20 |  | 455 |  |  |  |  |  |  |  |
| **EXC** | 45 | 1 | 4 | 1349 |  |  |  |  |  |  |
| **GOL** | 23 | 3 | 55 | 23 | 228 |  |  |  |  |  |
| **LYS** | 3 |  |  | 10 | 1 | 89 |  |  |  |  |
| **MIT** | 53 |  | 12 | 10 | 4 |  | 643 |  |  |  |
| **NUC** | 735 | 4 | 5 | 34 | 1 |  | 19 | 4128 |  |  |
| **PLA** | 119 | 3 | 5 | 105 | 15 |  | 12 | 37 | 2949 |  |
| **POX** | 8 |  |  |  |  |  | 1 |  |  | 70 |

**MOSQUITO (*A.gambiae*)**

Table 14 – Subcellular proteome percentage estimates for mosquito

|  | **CYT** | **CSK** | **END** | **EXC** | **GOL** | **LYS** | **MIT** | **NUC** | **PLA** | **POX** |
| --- | --- | --- | --- | --- | --- | --- | --- | --- | --- | --- |
| **CYT** | 14.43 |  |  |  |  |  |  |  |  |  |
| **CSK** | 0.25 | 1.11 |  |  |  |  |  |  |  |  |
| **END** | 0.10 |  | 3.25 |  |  |  |  |  |  |  |
| **EXC** | 0.32 |  | 0.06 | 14.28 |  |  |  |  |  |  |
| **GOL** | 0.06 | 0.01 | 0.21 | 0.03 | 1.07 |  |  |  |  |  |
| **LYS** | 0.01 |  |  | 0.02 | 0.02 | 0.53 |  |  |  |  |
| **MIT** | 0.40 |  | 0.02 | 0.10 | 0.01 |  | 5.52 |  |  |  |
| **NUC** | 3.44 | 0.05 | 0.02 | 0.66 | 0.05 |  | 0.08 | 29.50 |  |  |
| **PLA** | 0.38 | 0.05 | 0.05 | 1.34 | 0.07 |  | 0.13 | 0.48 | 21.36 |  |
| **POX** | 0.02 |  |  |  |  |  | 0.01 |  |  | 0.48 |

Table 15 – Subcellular proteome numeric estimates for mosquito

|  | **CYT** | **CSK** | **END** | **EXC** | **GOL** | **LYS** | **MIT** | **NUC** | **PLA** | **POX** |
| --- | --- | --- | --- | --- | --- | --- | --- | --- | --- | --- |
| **CYT** | 2140 |  |  |  |  |  |  |  |  |  |
| **CSK** | 38 | 164 |  |  |  |  |  |  |  |  |
| **END** | 15 |  | 483 |  |  |  |  |  |  |  |
| **EXC** | 48 |  | 9 | 2118 |  |  |  |  |  |  |
| **GOL** | 9 | 2 | 31 | 5 | 159 |  |  |  |  |  |
| **LYS** | 2 |  |  | 3 | 3 | 79 |  |  |  |  |
| **MIT** | 60 |  | 3 | 15 | 2 |  | 818 |  |  |  |
| **NUC** | 510 | 7 | 3 | 98 | 7 |  | 12 | 4376 |  |  |
| **PLA** | 56 | 7 | 7 | 199 | 10 |  | 19 | 72 | 3169 |  |
| **POX** | 3 |  |  |  |  |  | 2 |  |  | 72 |

**FRUITFLY (*D.melanogaster*)**

Table 16 – Subcellular proteome percentage estimates for fruitfly

|  | **CYT** | **CSK** | **END** | **EXC** | **GOL** | **LYS** | **MIT** | **NUC** | **PLA** | **POX** |
| --- | --- | --- | --- | --- | --- | --- | --- | --- | --- | --- |
| **CYT** | 12.74 |  |  |  |  |  |  |  |  |  |
| **CSK** | 0.24 | 1.05 |  |  |  |  |  |  |  |  |
| **END** | 0.06 |  | 2.85 |  |  |  |  |  |  |  |
| **EXC** | 0.22 | 0.01 | 0.02 | 12.26 |  |  |  |  |  |  |
| **GOL** | 0.07 | 0.03 | 0.12 | 0.04 | 1.40 |  |  |  |  |  |
| **LYS** | 0.01 |  |  | 0.07 | 0.01 | 0.55 |  |  |  |  |
| **MIT** | 0.32 |  | 0.02 | 0.12 |  |  | 4.86 |  |  |  |
| **NUC** | 2.76 | 0.03 | 0.01 | 0.37 | 0.02 |  | 0.09 | 37.38 |  |  |
| **PLA** | 0.28 | 0.01 | 0.02 | 0.88 | 0.05 |  | 0.07 | 0.42 | 20.06 |  |
| **POX** |  |  |  |  |  |  | 0.01 |  |  | 0.42 |

Table 17 – Subcellular proteome numeric estimates for fruitfly

|  | **CYT** | **CSK** | **END** | **EXC** | **GOL** | **LYS** | **MIT** | **NUC** | **PLA** | **POX** |
| --- | --- | --- | --- | --- | --- | --- | --- | --- | --- | --- |
| **CYT** | 1682 |  |  |  |  |  |  |  |  |  |
| **CSK** | 35 | 139 |  |  |  |  |  |  |  |  |
| **END** | 11 |  | 377 |  |  |  |  |  |  |  |
| **EXC** | 30 | 1 | 7 | 1619 |  |  |  |  |  |  |
| **GOL** | 7 | 1 | 23 | 7 | 184 |  |  |  |  |  |
| **LYS** | 1 |  |  | 11 | 3 | 72 |  |  |  |  |
| **MIT** | 49 |  | 1 | 11 |  |  | 642 |  |  |  |
| **NUC** | 405 | 7 | 3 | 67 | 4 |  | 11 | 4936 |  |  |
| **PLA** | 38 | 3 | 3 | 132 | 7 |  | 10 | 51 | 2649 |  |
| **POX** | 1 |  |  |  |  |  | 1 |  |  | 56 |

**WORM (*C.elegans*)**

Table 18 – Subcellular proteome percentage estimates for worm

|  | **CYT** | **CSK** | **END** | **EXC** | **GOL** | **LYS** | **MIT** | **NUC** | **PLA** | **POX** |
| --- | --- | --- | --- | --- | --- | --- | --- | --- | --- | --- |
| **CYT** | 14.80 |  |  |  |  |  |  |  |  |  |
| **CSK** | 0.20 | 1.19 |  |  |  |  |  |  |  |  |
| **END** | 0.08 |  | 3.47 |  |  |  |  |  |  |  |
| **EXC** | 0.23 | 0.02 | 0.07 | 12.60 |  |  |  |  |  |  |
| **GOL** | 0.09 | 0.02 | 0.11 | 0.05 | 1.31 |  |  |  |  |  |
| **LYS** |  |  |  | 0.03 | 0.01 | 0.58 |  |  |  |  |
| **MIT** | 0.25 |  | 0.02 | 0.08 |  |  | 5.84 |  |  |  |
| **NUC** | 2.36 | 0.04 |  | 0.39 | 0.01 |  | 0.06 | 29.75 |  |  |
| **PLA** | 0.20 | 0.03 | 0.04 | 0.60 | 0.08 |  | 0.05 | 0.24 | 24.41 |  |
| **POX** | 0.03 |  |  |  |  |  | 0.01 |  |  | 0.66 |

Table 19 – Subcellular proteome numeric estimates for worm

|  | **CYT** | **CSK** | **END** | **EXC** | **GOL** | **LYS** | **MIT** | **NUC** | **PLA** | **POX** |
| --- | --- | --- | --- | --- | --- | --- | --- | --- | --- | --- |
| **CYT** | 3147 |  |  |  |  |  |  |  |  |  |
| **CSK** | 44 | 252 |  |  |  |  |  |  |  |  |
| **END** | 16 |  | 738 |  |  |  |  |  |  |  |
| **EXC** | 49 | 4 | 15 | 2679 |  |  |  |  |  |  |
| **GOL** | 20 | 4 | 24 | 11 | 279 |  |  |  |  |  |
| **LYS** |  |  |  | 5 | 2 | 123 |  |  |  |  |
| **MIT** | 53 |  | 4 | 16 |  |  | 1242 |  |  |  |
| **NUC** | 502 | 9 |  | 83 | 2 |  | 13 | 6325 |  |  |
| **PLA** | 44 | 7 | 9 | 127 | 16 |  | 11 | 51 | 5190 |  |
| **POX** | 7 |  |  |  |  |  | 2 |  |  | 140 |

**YEAST (*S.cerevisiae*)**

Table 20 – Subcellular proteome percentage estimates for yeast

|  | **CYT** | **CSK** | **END** | **EXC** | **GOL** | **LYS** | **MIT** | **NUC** | **PLA** | **POX** |
| --- | --- | --- | --- | --- | --- | --- | --- | --- | --- | --- |
| **CYT** | 15.22 |  |  |  |  |  |  |  |  |  |
| **CSK** | 0.28 | 1.07 |  |  |  |  |  |  |  |  |
| **END** | 0.06 |  | 2.71 |  |  |  |  |  |  |  |
| **EXC** | 0.09 |  |  | 8.88 |  |  |  |  |  |  |
| **GOL** | 0.11 |  | 0.43 |  | 1.48 |  |  |  |  |  |
| **LYS** |  |  |  | 0.02 |  | 0.11 |  |  |  |  |
| **MIT** | 0.77 | 0.02 | 0.09 | 0.11 |  |  | 9.55 |  |  |  |
| **NUC** | 6.49 | 0.17 | 0.19 | 0.21 | 0.02 |  | 0.30 | 33.53 |  |  |
| **PLA** | 0.13 |  | 0.04 | 0.50 | 0.04 |  | 0.09 | 0.47 | 16.19 |  |
| **POX** | 0.06 |  |  |  |  |  | 0.04 |  |  | 0.54 |

Table 21 – Subcellular proteome numeric estimates for yeast

|  | **CYT** | **CSK** | **END** | **EXC** | **GOL** | **LYS** | **MIT** | **NUC** | **PLA** | **POX** |
| --- | --- | --- | --- | --- | --- | --- | --- | --- | --- | --- |
| **CYT** | 860 |  |  |  |  |  |  |  |  |  |
| **CSK** | 16 | 60 |  |  |  |  |  |  |  |  |
| **END** | 3 |  | 153 |  |  |  |  |  |  |  |
| **EXC** | 5 |  |  | 502 |  |  |  |  |  |  |
| **GOL** | 6 |  | 24 |  | 84 |  |  |  |  |  |
| **LYS** |  |  |  | 1 |  | 6 |  |  |  |  |
| **MIT** | 43 | 1 | 5 | 6 |  |  | 540 |  |  |  |
| **NUC** | 367 | 10 | 11 | 12 | 1 |  | 17 | 1895 |  |  |
| **PLA** | 7 |  | 2 | 29 | 2 |  | 5 | 26 | 915 |  |
| **POX** | 3 |  |  |  |  |  | 2 |  |  | 31 |
